# Analysis of Differentially Expressed Long Non-coding RNAs and the Associated TF-mRNA Network in Tongue Squamous Cell Carcinoma

**DOI:** 10.3389/fonc.2020.01421

**Published:** 2020-08-14

**Authors:** Mi Zhang, Zexi Chen, Sihui Zhang, Ling Wu, Yinghui Jie, Yunyang Liao, Yue Huang, Jiang Chen, Bin Shi

**Affiliations:** ^1^School and Hospital of Stomatology, Fujian Medical University, Fuzhou, China; ^2^Fujian Key Laboratory of Oral Diseases, School and Hospital of Stomatology, Fujian Medical University, Fuzhou, China; ^3^Research Center of Dental and Craniofacial Implants, School and Hospital of Stomatology, Fujian Medical University, Fuzhou, China; ^4^Department of Oral and Maxillofacial Surgery, The First Affiliated Hospital of Fujian Medical University, Fuzhou, China

**Keywords:** tongue squamous cell carcinomas, lncRNA, mRNA, transcription factor, network

## Abstract

Accumulating evidence indicates that long non-coding RNAs (lncRNAs) play crucial roles in tongue squamous cell carcinoma (TSCC) tumorigenesis. However, the comprehensive regulation of lncRNAs-transcription factors (TFs)-messenger RNAs (mRNAs) in TSCC remains largely unknown. The purpose of this study was to identify aberrantly expressed lncRNAs and the associated TF-mRNA network in TSCC. To explore lncRNA and mRNA expression profiles and their biological functions in TSCC, we surveyed the lncRNA and mRNA expression profiles of TSCC and adjacent tissues using next-generation RNA sequencing in six patients. Thousands of significantly differentially expressed lncRNAs (DELs) and mRNAs (DEGs) were identified. Gene Ontology (GO) and Kyoto Encyclopedia of Genes and Genomes (KEGG) pathway enrichment analyses were performed to demonstrate the principal functions of the significantly dysregulated lncRNAs and genes. A total of 40 DELs were screened between TSCC and adjacent non-cancerous tissues. Results obtained from GEPIA and StarBase confirmed the expression levels of nine pivotal DELs obtained in our study. Three of the nine deregulated DELs were identified to have a significant impact on the overall survival of TSCC patients, which were evaluated with GEPIA and StarBase. LncMAP was used to predict the lncRNA-TF-mRNA triplets in TSCC. Furthermore, based on these results, we established lncRNA-TF-mRNA coexpression networks for the up- and downregulated lncRNAs using Cytoscape. We also found that among the nine pivotal lncRNAs, there is limited research on the abnormally expressed lncRNAs, including RP11-54H7.4, CTD-2545M3.8, RP11-760H22.2, RP4-791M13.3, and LINC01405, in TSCC pathogenesis. This is the first study to show that RP11-54H7.4, LINC00152, and LINC01405 can be acted as a prognostic target for TSCC. Our findings provide a novel perspective and lay the foundation for future research on the potential roles of lncRNAs, TFs, and mRNAs in TSCC. Verification of the potential lncRNA-TF-mRNA regulatory networks will provide a more comprehensive understanding of the pathogenesis of TSCC.

## Introduction

As the most lethal and commonly occurring oral malignancy, oral squamous cell carcinoma (OSCC) accounts for 95% of all oral cancer diagnoses and causes more than 500,000 deaths per year (1). Approximately 25–40% of OSCCs are diagnosed as tongue squamous cell carcinoma (TSCC) (2). TSCC is characterized by frequent lymphoid metastasis, a high rate of regional recurrence, and a poor prognosis. Moreover, despite great progress in surgical techniques, diagnostic procedures, chemotherapy, and radiotherapy, the 5-year overall survival rate of TSCC patients has not improved significantly over the past decades. Therefore, a better understanding of the molecular mechanisms behind the initiation and progression of TSCC may facilitate the diagnosis and treatment of this malignant tumor.

Long non-coding RNA (lncRNA) represents a class of RNA species that are transcribed predominantly by RNA polymerase II, with a length exceeding 200 nucleotides and no apparent protein-coding potential, as indicated by the lack of strongly translated open reading frames (ORFs) (3). lncRNA plays a crucial role in diverse biological systems through genomic imprinting, cell cycle regulation, and cell differentiation and has been linked to a number of human diseases, especially cancer (4, 5). Recently, growing evidence has demonstrated that lncRNAs may also have essential roles in TSCC. For example, overexpression of the lncRNA FALEC represses malignant behaviors in TSCC (6). Upregulation of the lncRNA HOTTIP in TSCC patients indicates a poor clinical prognosis (7). In TSCC cell lines, cell growth, invasion, and migration abilities are associated with the overexpression of MALAT-1 and AFAP1-AS1 via the Wnt signaling pathway, while the knockdown of MALAT-1 in TSCC cells leads to the upregulation of certain SPRR proteins (8–10). High expression of the lncRNA UCA1 has been linked to the migratory ability of epithelial cancer cells and regional lymph node metastasis in TSCC (11). Moreover, the lncRNAs KCNQ1OT1 and SNHG17 act as competing endogenous RNAs (ceRNAs) to regulate cell proliferation and cancer progression in TSCC (12, 13).

In our study, we analyzed the expression profiles of lncRNAs and mRNAs in TSCC tissue through high-throughput sequencing. In addition, we identified molecular function, cellular component, biological process, and pathway analyses enriched by mRNA-associated lncRNAs and differentially expressed mRNAs in TSCC. Furthermore, our work revealed several pivotal lncRNAs related to the overall survival of TSCC patients. Finally, to optimize the regulatory mechanism of lncRNAs, we further explored several reliable transcription factors (TFs) in the regulatory regions of lncRNAs and constructed a lncRNA-TF-mRNA coexpression network.

## Materials and Methods

### Clinical Samples and Data Processing

Tumors and adjacent tongue tissues were obtained from patients with TSCC who underwent surgery at The First Affiliated Hospital of Fujian Medical University. Following surgical resection, six pairs of tissue samples were immediately immersed in liquid nitrogen and then stored at −80°C. Written informed consent was obtained from all participants in accordance with the Declaration of Helsinki, and the study protocol was approved by the Ethics Committee of The First Affiliated Hospital of Fujian Medical University (Fuzhou, China).

Next-generation RNA sequencing assays were performed to detect the mRNA and lncRNA expression profiles by KangChen Biotech (Shanghai, China) using an Illumina HiSeq 4,000 system (Illumina, San Diego, CA, USA). Solexa pipeline version 1.8 was used to align the reads to the genome, generate raw counts corresponding to each known gene (a total of 17,242 genes), and calculate the reads per kilobase per million (RPKM) values. The differentially expressed lncRNAs (DELs) and mRNAs (DEGs) were identified through fold change/*p*-value/FDR filtering [fold change ≥1.5, *p* < 0.05, and FDR (adjusted *p*-value) < 0.05].

Then, the data associated with TSCC were also retrieved from The Cancer Genome Atlas (TCGA) database (https://portal.gdc.cancer.gov/) and were downloaded through GDC data transfer tool, including the RNA sequencing (RNA-Seq) of transcriptome profiling and clinical data. Then, in the predictive survival related model, we excluded 27 samples because they lacked complete clinical data. Finally, 146 TSCC samples and 15 normal control samples were collected in our study. EdgeR package in R (version 3.6.3) was used to identify the DELs. After that, we used the annotation file in GTF format (Homo_sapiens.GRCh38.100.chr.gtf) to identify and annotate DELs with the thresholds of |log2FC|>2.0 and FDR < 0.05.

### Cell Lines

Three human TSCC cell lines, CAL-27, SCC-9, and SCC-25 were purchased from the American Type Culture Collection (ATCC; Manassas, VA, USA), and the human normal oral keratinocyte (hNOK) cell line was obtained from the Institute of Biochemistry and Cell Biology of the Chinese Academy of Sciences (Shanghai, China). CAL-27 cells and hNOK cells were grown in DMEM (Gibco) supplemented with 10% FBS (Invitrogen, Carlsbad, CA, USA). SCC-9 and SCC-25 cells were cultured in DMEM/F-12 (Gibco) supplemented with 10% FBS and 0.4 μg/ml hydrocortisone. All cells were maintained at 37°C in an incubator supplied with 5% CO_2_.

### RNA Extraction and Quantitative Real-Time PCR Analysis

Total RNA was extracted from tissues or cells using TRIzol reagent (Invitrogen, CA, USA) according to the manufacturer's instructions. Total RNA was reverse transcribed into cDNA using a PrimeScript RT reagent kit (TaKaRa). RT-qPCR analyses were performed using SYBR Green Master Mix (TaKaRa). This process was performed using an Applied Biosystems 7,500 Real-Time PCR System. The results were normalized to the expression of glyceraldehyde-3-phosphate dehydrogenase (GAPDH). The relative expression level was calculated by the 2^−ΔΔ^ Ct method. The primers were provided by the SunYa Company.

### Filtering DELs and DEGs

DELs and DEGs were selected using the following cut-off criteria: for DELs: the top 20 upregulated and downregulated lncRNAs, for DEGs: |log2-fold change| >2 and *p* < 0.05. Heatmap charts and volcano plots were established the gplots package in R software.

### Verification of DEL and DEG Expression in GEPIA and StarBase

To identify the DELs, we used GEPIA (http://gepia.cancer-pku.cn/detail.php) (14) and StarBase (http://starbase.sysu.edu.cn/) (15), publicly available online databases, to explore gene expression levels in tumors and normal tissues.

### Gene Set Enrichment Analysis (GSEA)

GSEA is a calculation method used to determine whether a given gene set is significantly different between different groups. The genes in these gene sets have a certain degree of correlation (e.g., the same biological function, located close to each other on the chromosome or defined by themselves according to a certain standard). However, LncRNA can not be directly used for GSEA as mRNA, but the correlation between each DEL and all mRNAs can be calculated. Then, GSEA of DELs can be performed using GSEAPreranked in GSEA software.

The Gene Ontology (GO) project provides a controlled vocabulary to describe gene and gene products attributed in any organism (http://www.geneontology.org). GO covers three domains: biological process, cellular component, and molecular function. The lower the *p*-value is, the more significant the GO term enrichment among the differentially expressed genes (a *p* ≤ 0.05 is recommended). Pathway analysis is a functional analysis that maps genes to Kyoto Encyclopedia of Genes and Genomes (KEGG) pathways (http://www.genome.jp/kegg/). The *p*-value (EASE score, Fisher's *p*-value, or hypergeometric *p*-value) denotes the significance of the pathway correlated to the condition. The GO and KEGG pathway analyses were performed by KangChen Biotech (Shanghai, China).

### Survival Analysis of the Candidate lncRNAs

The GEPIA and StarBase databases were used to evaluate the effects of DELs on the overall survival of patients with TSCC. The final lncRNA correlated with overall survival was thus regarded as the pivotal lncRNA. *p* < 0.05 was considered statistically significant using the log-rank test. The cut-off value between two groups was set as the ‘‘median.''

### Establishing the lncRNA-TF-mRNA Network

LncMAP (http://bio-bigdata.hrbmu.edu.cn/LncMAP/index.jsp) was used to analyze the lncRNA-TF-mRNA triplets in TSCC. A Venn diagram was drawn to determine the intersection between the mRNA in triplets and DEGs. The expression levels of these overlapping mRNAs were validated with GEPIA, and the correlation of the TF and mRNA was analyzed with StarBase. On the basis of these findings, we constructed a lncRNA-TF-mRNA regulatory network using Cytoscape (version 3.7.2) software to visualize their interactions. lncRNA-TF-mRNA triplets that showed no correlations with other triplets were excluded from the network.

### Identification of the Prognostic Biomarkers

After analyzing the effects of pivotal DELs on the overall survival of patients with TSCC, we verified our findings in the TCGA difference analysis results, and it confirmed that LINC00152, LINC01405, RP11-54H7.4, and RP11-760H22.2 were simultaneously identified to be differentially expressed in TSCC. Thus, we also made the expression scatter and pairing plots of this four pivotal lncRNA by R software.

### Statistical Analysis

Data are presented as the mean ± standard error (SE). Measured data were compared between groups using Student's paired *t*-tests or independent *t*-test. All statistical analyses were performed using SPSS and R software (version 22; IBM, Armonk, NY, USA) and presented graphically in GraphPad Prism 5.0 (GraphPad, La Jolla, CA, USA). Making the scatter diagram and paired plot by wilcoxon test in R software. All tests were two-tailed, and *p* < 0.05 was considered statistically significant.

## Results

### Identification of the Candidate DELs and DEGs in TSCC

Tumor and adjacent tissue samples were collected from six patients with TSCC. The main clinical characteristics of the enrolled patients are summarized in Supplementary Table 1. After sequencing, a total of 51,349 transcripts were presented, of which 38,567 are involved in protein coding. Among these transcripts, 4,224 mRNAs and 1,470 lncRNAs were differentially expressed between TSCC tumor and adjacent tissues. Among the mRNAs, 2,792 were upregulated and 1,432 were downregulated. Among the lncRNAs identified, 947 were upregulated and in TSCC and 523 were downregulated in TSCC (*p* < 0.05).

**Figure 1:**
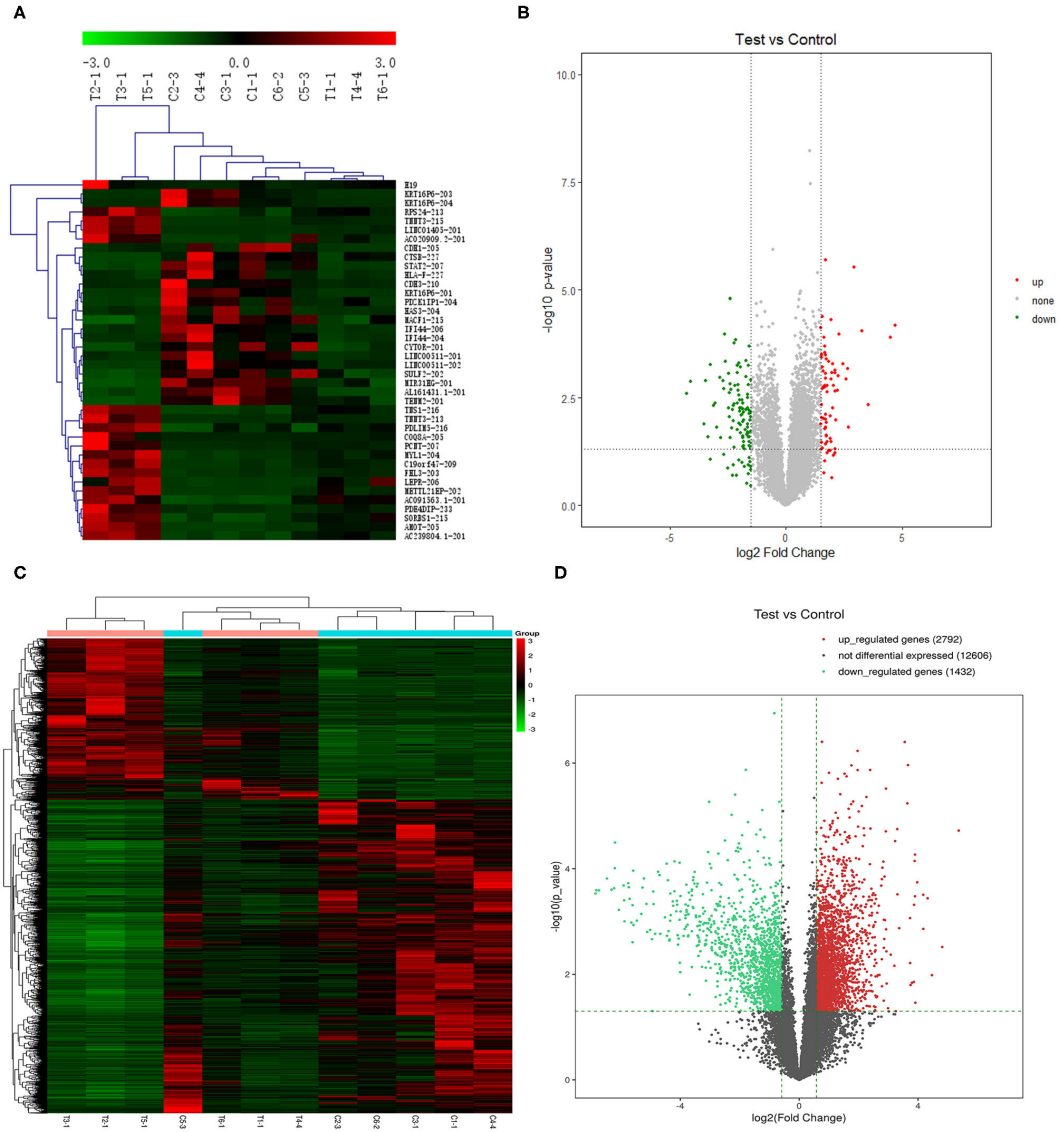
Heatmap plot of **(A)** 20 upregulated lncRNAs and 20 downregulated DELs and **(C)** the significantly expressed mRNAs. Volcano plot of **(B)** the significantly expressed lncRNAs and **(D)** mRNAs (*p* < 0.05, fold change >1.5). Synonyms: RP11-54H7.4 (AL161431.1-201), LINC00152 (CYTOR-201), RP11-760H22.2 (AC091563.1-201), CTD-2545M3.8 (AC020909.2-201), and RP4-791M13.3 (AC239804.1-201).

We further screened the 40 DELs and 450 DEGs that were aberrantly expressed between all TSCC tumor and adjacent tissues. As shown in Figure 1, the heatmap plot of (A) the top 20 upregulated and downregulated DELs and (B) all significantly expressed mRNAs were shown. Moreover, the volcano plot of (C) the top 20 upregulated and downregulated DELs and (D) all significantly expressed mRNAs were constructed. The annotation of these DELs is summarized in Table 1. Information on the 450 DEGs is presented in Table 2.

**Table 1:** Information on 40 DELs in TSCC.

**Category**	**LncRNA symbol**	**Ensembl ID**	**Locus**	**log_**2**_FC**	***p*-value**
Upregulated	KRT16P6-203	ENST00000583748	chr17:16722827-16725442	4.6923889	6.564E-05
	KRT16P6-204	ENST00000584481	chr17:16721306-16722677	4.494188	0.0001269
	CTSB-227	ENST00000533110	chr8:11705188-11707556	3.5230094	0.0047111
	KRT16P6-201	ENST00000417510	chr17:16721297-16725893	3.2691007	8.906E-05
	RP11-54H7.4	ENST00000618966	chr13:109921982-109926186	2.9241856	3.041E-06
	CDH1-205	ENST00000562836	chr16:68797197-68867441	2.672898	0.0157189
	MIR31HG-201	ENST00000304425	chr9:21455641-21559668	2.6329908	0.0006836
	IFI44-206	ENST00000472152	chr1:79115543-79129763	2.5667645	0.0011736
	STAT2-207	ENST00000556539	chr12:56735381-56743559	2.2608986	0.0001073
	LINC00152	ENST00000331944	chr2:87754948-87821037	2.2235254	0.0010058
	CDH3-210	ENST00000569117	chr16:68730047-68730826	2.219285	0.0021646
	PDZK1IP1-204	ENST00000491793	chr1:47649272-47651016	2.1330875	0.0106663
	LINC00511-201	ENST00000453722	chr17:70380443-70588943	2.0909352	0.0022785
	IFI44-204	ENST00000467790	chr1:79115527-79121196	2.0872088	0.0008379
	LINC00511-202	ENST00000457958	chr17:70399463-70588479	2.0860305	0.0007322
	HAS3-204	ENST00000568321	chr16:69140157-69140891	2.0859456	0.0488404
	MACF1-215	ENST00000473843	chr1:39900157-39901679	2.0165445	0.0016772
	HLA-F-227	ENST00000485513	chr6:29691717-29693076	1.9916707	0.0055367
	SULF2-202	ENST00000433632	chr20:46290178-46292298	1.9859734	0.0005177
	TENM2-201	ENST00000517586	chr5:167248073-167379660	1.949576	0.0008307
Downregulated	TNNT3-215	ENST00000639560	chr11:1949052-1959713	−4.262896	0.0025895
	LINC01405-201	ENST00000331096	chr12:111374406-111375255	−4.084115	0.0013337
	H19-212	ENST00000446406	chr11:2016446-2019057	−3.534739	0.0131478
	TNS1-216	ENST00000480665	chr2:218713195-218723356	−3.447791	0.0013045
	RPS24-213	ENST00000485708	chr10:79793602-79800428	−3.09478	0.0048687
	TNNT3-213	ENST00000492075	chr11:1946148-1955885	−2.969187	0.0151812
	CTD-2545M3.8	ENST00000595005	chr19:50990067-50990895	−2.809795	0.0268764
	MYL1-204	ENST00000496436	chr2:211155756-211168339	−2.693001	0.0012879
	C19orf47-209	ENST00000584868	chr19:40826987-40828246	−2.678462	0.0019579
	AMOT-205	ENST00000462114	chrX:112066365-112083911	−2.629755	0.0001059
	PCNT-207	ENST00000483844	chr21:47768153-47773240	−2.435337	0.0086552
	METTL21EP-202	ENST00000605100	chr13:103532449-103548383	−2.402461	0.0015185
	COQ8A-205	ENST00000478406	chr1:227127995-227175244	−2.383483	0.0264686
	PDLIM5-216	ENST00000511767	chr4:95376418-95471137	−2.286661	0.006016
	FHL3-203	ENST00000477194	chr1:38463017-38471278	−2.264287	0.0049033
	RP11-760H22.2	ENST00000520544	chr8:121064419-121068423	−2.21687	0.0001699
	PDE4DIP-233	ENST00000533845	chr1:144946702-144994925	−2.201192	0.0016894
	LEPR-206	ENST00000462765	chr1:65991372-66075951	−2.079886	0.0005774
	SORBS1-215	ENST00000474353	chr10:97101341-97321130	−2.077141	0.0008195
	RP4-791M13.3	ENST00000466343	chr1:144833168-144835867	−2.071663	0.0015749

**Table 2:** Information on DEGs in TSCC.

**Category**	**Gene symbol**
Upregulated	MMP1, S100A7, PI3, MMP10, KRT17, LAMC2, COL1A1, KRTDAP, SERPINE1, S100A7A, CDH3, KLK5, KRT6B, CXCL10, KRT16, KRT14, ISG15, TYMP, MMP3, CXCL13, TNC, IFI30, MMP9, DEFB4A, TENM2, HAS3, MMP12, POSTN, IFI6, COL12A1, PLAU, GBP1, FSCN1, BNC1, ADAM12, COL3A1, CXCL9, PTHLH, LAMP3, KLK7, ADAMTS2, GBP5, KLK6, INHBA, TNS4, S100A12, SAMD9, MMP11, SERPINB4, OAS2, CTHRC1, S100A2, BST2, THBS2, CLCA2, DDX60, OAS3, KRT5, CXCL1, CPA4, COL5A1, CTSC, COL1A2, GJB6, OASL, MFAP2, EPSTI1, PYGL, TAP1, NELL2, IFI44, PTK7, COL7A1, SLC2A1, CPXM1, WFDC5, LAMB3, NME1-NME2, SCO2, COL5A2, RAB31, MYO1B, ACP5, MMP7, UBE2L6, RSAD2, RBP1, VMP1, IFIT3, PLEK2, GJA1, EPPK1, CASP14, FAT1, MX1, IGF2BP2, FAM83A, TGFBI, DNAH17, SPP1, CALML5, UBD, TTYH3, PARP14, MYBL2, FAM83B, THBS1, CLDN1, FBLIM1, UCN2, WARS, HELZ2, XAF1, TREM2, PDZK1IP1, STAT1, FABP5, ITGA3, CDC25B, TLR2, ADAMTS14, HIST1H2BO, ARNTL2, IFI27, CDC20, FAT2, WISP1, FCGR3A, LOXL2, MMP14, AIM2, IFI44L, GSDMC, GPR68, DDX58, IL12RB2, IFIH1, SULF2, GPRIN1, BASP1, MARCKSL1, RTP4, CRABP2, ENTPD7, HIST1H2AL, WNT7B, ACTN1, THY1, HIF1A, C6orf141, CTSV, HIST1H2AM, SOCS3, PLA2G7, COL4A1, STAT2, TPBG, SLC7A5, TPX2, APOBEC3A, IFFO2, PTGFRN, IFI16, ADAMTS12, SLC6A14
Downregulated	RORC, KLF15, LIFR, CAVIN2, PYGM, PAIP2B, VIT, CYP4B1, TLE2, CNTFR, SELENBP1, SLC25A4, PERM1, DEPTOR, HFE2, ATP2A1, CASQ1, FHL1, TNXB, LDB3, PPP1R3C, TNNC2, ENO3, PI16, PPARGC1A, FBXO40, CKMT2, AMOT, ANKRD2, NRAP, SLC2A4, SH3BGR, PLIN4, SCARA5, HSPB6, AC131097.2, MYOZ1, PGAM2, MB, PPP1R3A, MYH7, MYH2, CKM, MLIP, SPTB, RPL3L, EEF1A2, XIRP1, ATP1A2, ACTA1, ASB2, AMPD1, SRL, PRKAA2, MYBPC1, KLHL40, COX6A2, CA3, MYLK4, HIF3A, LDHD, CLEC3B, LMOD2, GDF10, MYOT, PEBP4, TCAP, PFKM, PADI2, MYOM1, GPT, MYH11, SYPL2, FXYD1, NFIX, CAND2, MYL2, SMTNL2, GAMT, SCN4A, SMYD1, COX7A1, LMOD1, TNNI2, FITM1, MYPN, SMTNL1, TRIM54, TMOD1, ANGPTL1, MYOZ2, AGBL1, CRYAB, TUBA8, SOD3, RRAGD, COQ8A, CMYA5, FABP3, MAOB, SORBS1, MYLK3, MLXIPL, ACACB, TPM2, DUSP13, NDRG2, MYLPF, RYR1, CHCHD10, MYL1, GPD1, LRRC2, ANKRD23, ART3, KLHL41, OBSCN, S100A1, PLN, NT5C1A, ADPRHL1, C10orf71, TTN, DUSP27, LRRC39, TMEM38A, SYNM, ABCA8, CACNA1S, AGL, SFRP1, RCAN2, CLIC5, SYNPO2, PHYH, HSPB7, MYBPC2, FNDC5, HRC, PLIN5, PLAC9, APOBEC2, MYOM2, CFD, NEB, ADSSL1, MYO18B, TCEA3, TNNT3, ACTN2, CAPN3, GPIHBP1, TNNT1, TMEM52, TRDN, PPP1R1A, MYL3, GOT1, STAC3, PDK4, SLC36A2, ADH1B, TXLNB, JPH1, FLNC, TNNC1, RTN2, FILIP1, SLN, GPC3, SPEG, MYH6, TMOD4, HSPB2, LMOD3, TMEM182, SMPX, DMD, CLCN4, DUSP26, AC083902.2, DES, ZBTB16, KLHL31, SNTA1, CHRDL1, ANO5, PHKG1, SGCA, AGT, RASD1, XIRP2, TRIM63, PKIA, MYF6, GRB14, CLCN1, SYNPO2L, MYH7B, NEURL1, USP13, CAMK2A, KLHL38, CAMK2B, JPH2, GMPR, UNC45B, LIMCH1, CALML6, MYOM3, HSPB8, KY, TRIM55, FHL3, ASB16, PDE4DIP, CACNG1, PLPP7, GADD45G, ACHE, ZNF106, JSRP1, ALDH1A1, PDLIM3, MYADML2, ASB5, AK1, BEST3, DYRK1B, PFKFB1, MYOZ3, ADH1C, NOS1, CSRP3, CFL2, TRIM72, SCN4B, SHISA4, DDIT4L, MYH14, PTGIS, MYLK2, CES1, ALPK3, TNNI1, TBX15, KLHL33, APOD, CAP2, PLA2G2A, CACNG6, PLIN1, TSPAN7, PGM1, SEMA6C, BIN1, MYH1, PRELP, RBM24, SH3BGRL2, CAV3, MFAP4, ANK1, STBD1, WNK2, PPP1R27, LPL, ADIPOQ, NEXN, NMRK2, CAVIN4, DPT, IP6K3, MGP, PRUNE2, CASQ2, HBA2, HBB, PODN, HSPB3, CILP, MYOG, MYH8, HBA1, KRT4

### Verification of the Expression of DELs and DEGs in Available Databases

**Figure 2:**
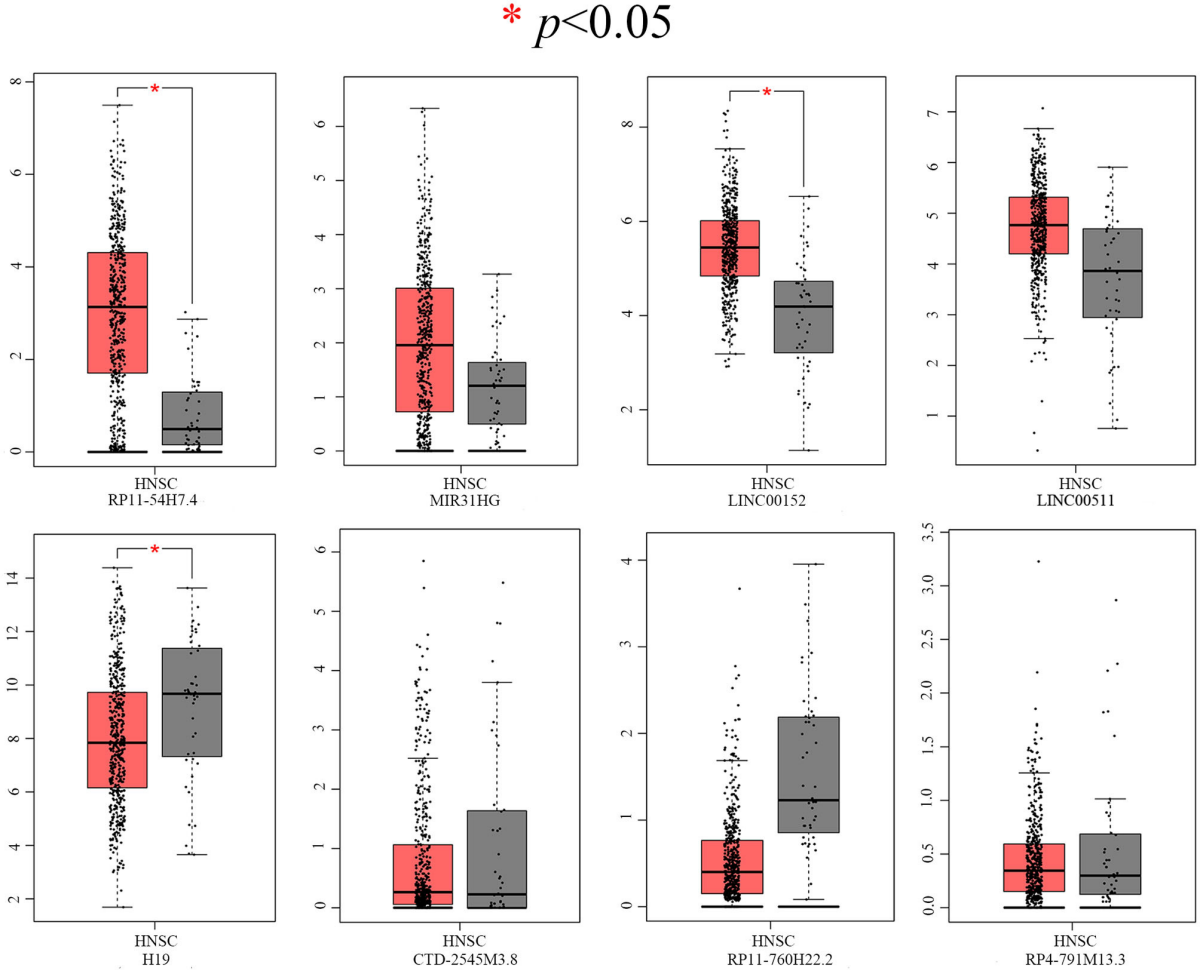
Verification of RP11-54H7.4, MIR31HG, LINC00152, LINC00511, H19, CTD-2545M3.8, RP11-760H22.2, and RP4-791M13.3 expression in HNSC by GEPIA. The red box represents expression in tumor tissue, and the gray box represents expression in normal tissue. HNSC, head, and neck squamous cell carcinoma (*p* < 0.05).

**Figure 3:**
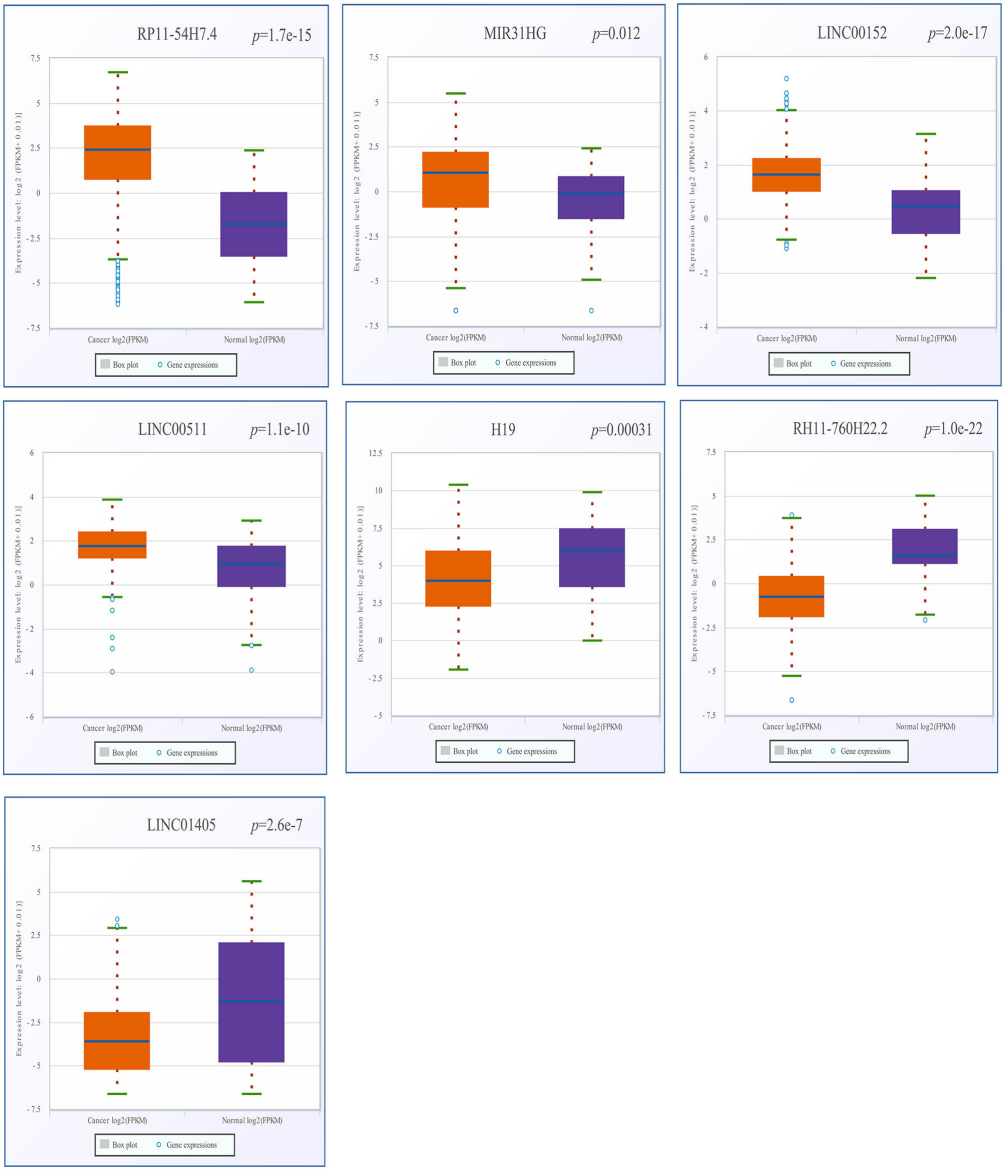
Verification of RP11-54H7.4, MIR31HG, LINC00152, LINC00511, H19, RP11-760H22.2, and LINC01405 expression in HNSC by StarBase. The orange box represents expression in tumor tissue, and the purple box represents expression in normal tissue. HNSC, head, and neck squamous cell carcinoma.

After screening, the expression profiles of the candidate DELs and DEGs were further validated with GEPIA and StarBase. We confirmed that nine keyDELs were aberrantly expressed between TSCC tumor and normal tissues. In GEPIA, with RP11-54H7.4, MIR31HG, LINC00152, and LINC00511 being upregulated and H19, CTD-2545M3.8, RP11-760H22.2, and RP4-791M13.3 being consistently downregulated in head and neck squamous cell carcinoma (HNSC) (Figure 2). Moreover, the results acquired in StarBase verified that the expression of the candidate DELs, such as RP11-54H7.4, MIR31HG, LINC00152, and LINC00511, was upregulated while the expression of H19, RP11-760H22.2, and LINC01405 was downregulated in HNSC (Figure 3).

### The GSEA Results of DELs

**Figure 4:**
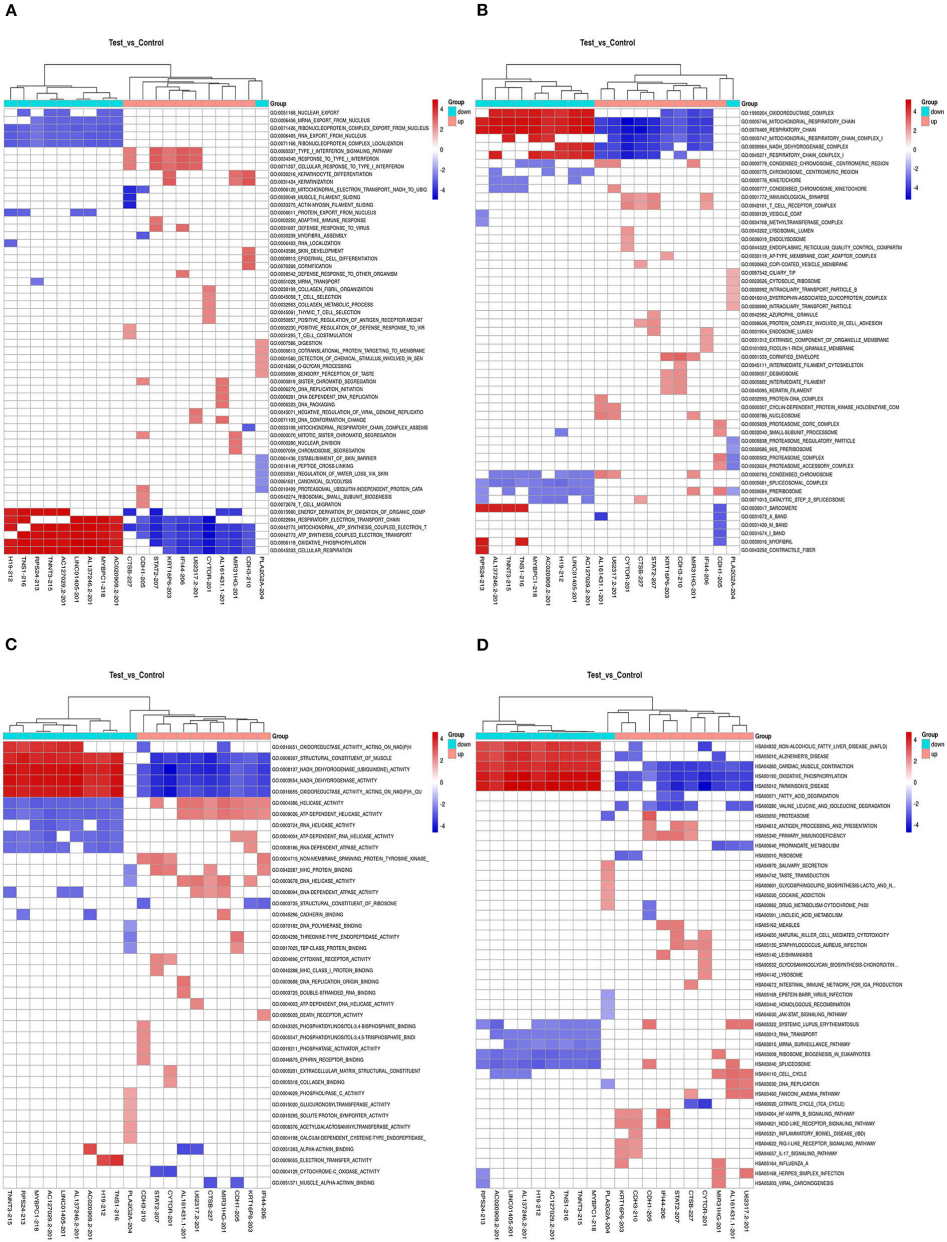
The GSEA results the pivotal DELs. **(A)** Biological processes of the DELs. **(B)** Cellular component of the DELs. **(C)** Molecular functions of the DELs. **(D)** Pathway analysis of the DELs.

We also calculated the correlation between each DEL and all mRNAs so that GSEA could be performed using GSEAPreranked in GSEA software. The biological process (Figure 4A), cellular component (Figure 4B), molecular function (Figure 4C), and pathway (Figure 4D) analyses of the pivotal DELs, including KRT16P6-203, CTSB-227, RP11-54H7.4-201, CDH1-205, MIR31HG-201, IFI44-206, STAT2-207, LINC00152-201, CDH3-210, TNNT3-215, LINC01405-201, H19-212, TNS1-216, RPS24-213, and CTD-2545M3.8, were performed. Our data showed that the biological processes associated with the aberrantly regulated lncRNAs were cellular respiration, oxidative phosphorylation, and ATP synthesis coupled electron transport. The cellular components associated with the aberrantly regulated lncRNAs were the oxidoreductase complex mitochondrial respiratory chain and respiratory chain. The molecular functions associated with the aberrantly regulated lncRNAs were NADH dehydrogenase activity and oxidoreductase activity. KEGG pathway enrichment analysis of the significant DELs was performed to understand gene-related pathways and molecular interactions. Our results showed that oxidative phosphorylation, RNA transport, and mRNA surveillance pathway were associated with the dysregulated lncRNAs. Meanwhile, the GSEA results of LINC00152, LINC01405, RP11-54H7.4, and RP11-760H22.2 were showed independently in Supplementary Table 2.

### Delineation of GO and KEGG Pathway Analyses of DEGs

**Figure 5:**
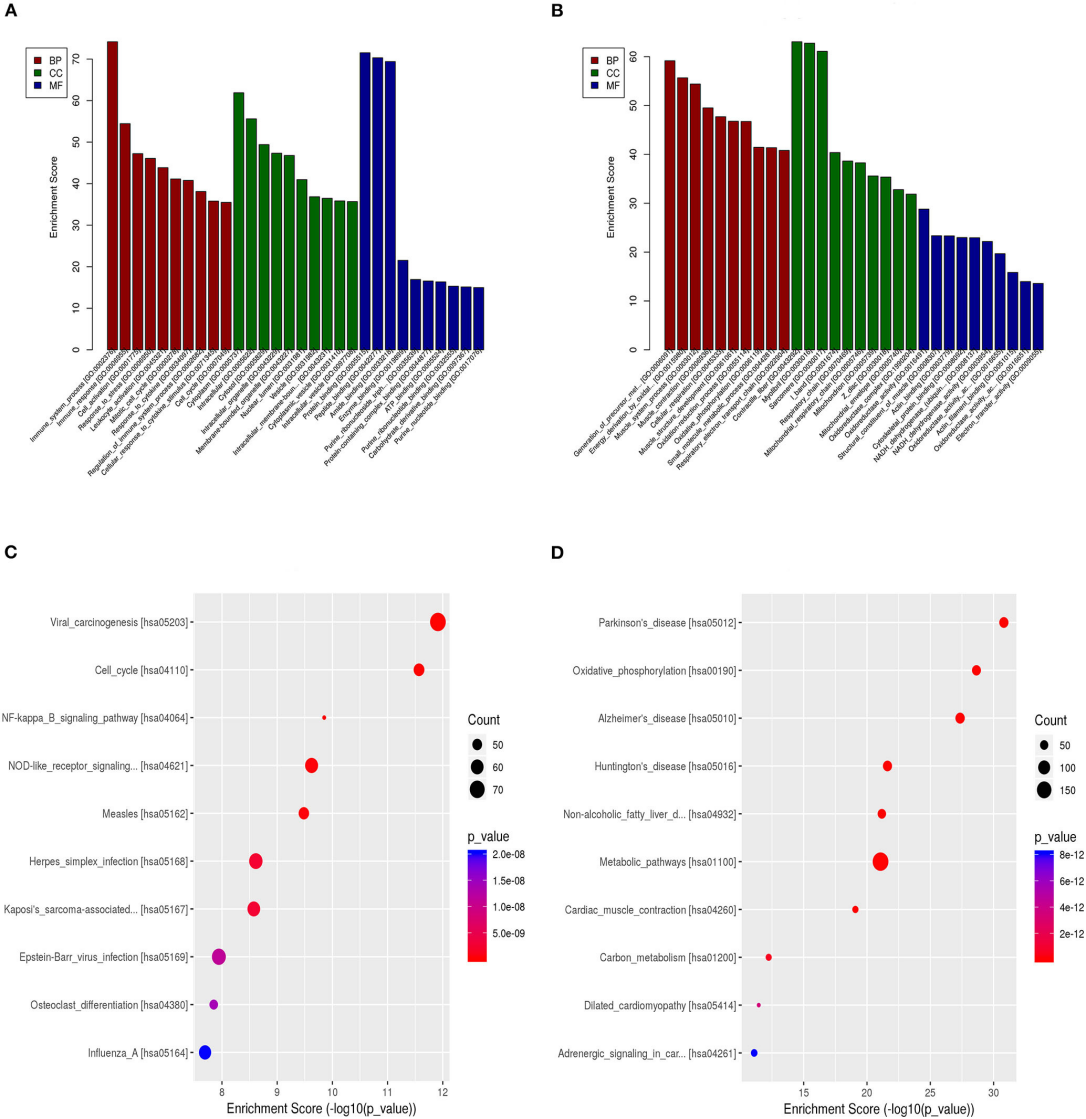
GO and pathway analyses of the DEGs. **(A)** GO annotation of upregulated mRNAs with the top 10 enrichment scores covering biological process, cellular component, and molecular function terms. **(B)** GO annotation of downregulated mRNAs with the top 10 enrichment scores covering biological process, cellular component, and molecular function terms. **(C)** Pathway analysis of upregulated DEGs. **(D)** Pathway analysis of downregulated DEGs.

GO enrichment analysis of the DEGs may reveal the roles of the significantly differentially regulated lncRNAs. Our data also showed that the biological processes associated with the upregulated mRNAs were immune system processes and the immune response (Figure 5A). The downregulated mRNAs were mostly enriched in the generation of precursor metabolites and energy, energy derivation by oxidation of organic compounds, and muscle system processes (Figure 5B). Regarding the cellular components, most of the upregulated mRNAs were related to the cytoplasm, intracellular and cytosol, and the downregulated mRNAs were mostly related to the contractile fiber, myofibril and sarcomere. Moreover, the molecular functions mainly associated with the upregulated mRNAs were protein binding, peptide binding, and amide binding, while those associated with the downregulated mRNAs were oxidoreductase activity, the structural constituent of muscle and actin binding. Importantly, KEGG pathway analysis of the DEGs revealed 20 pathways that could play pivotal roles in the mechanism of TSCC tumorigenesis, including the cell cycle, metabolic and NOD-like receptor signaling pathway (Figures 5C,D).

### Clinical Prognostic Significance of the Pivotal DELs for TSCC Patients

**Figure 6:**
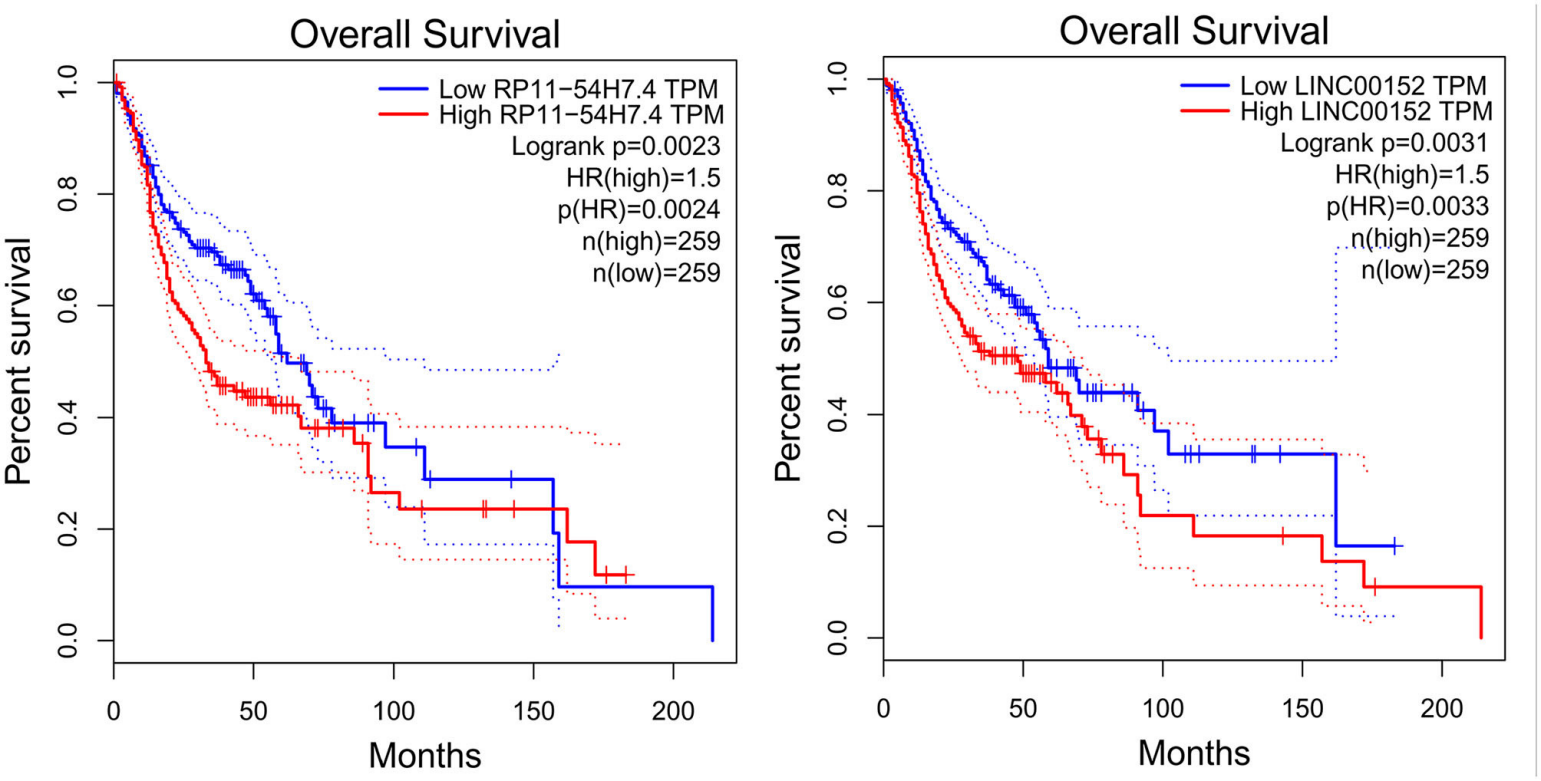
Clinical prognostic significance of RP11-54H7.4 and LINC00152 in HNSC by GEPIA. HNSC, head, and neck squamous cell carcinoma.

**Figure 7:**
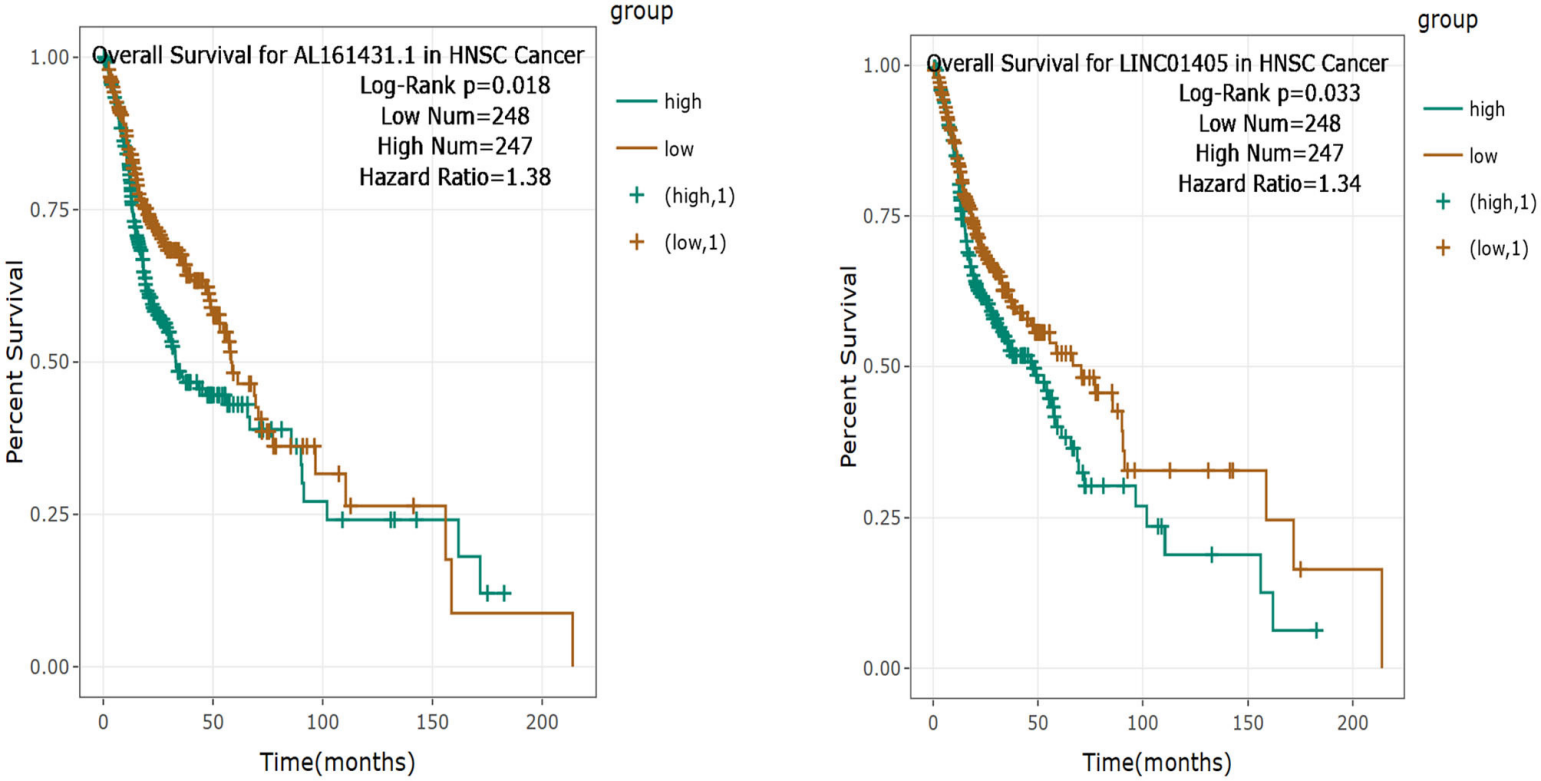
Clinical prognostic significance of RP11-54H7.4 and LINC01405 in HNSC by StarBase. HNSC, head, and neck squamous cell carcinoma.

Then, the significance of the nine key DELs on the overall survival of patients with TSCC was evaluated by GEPIA and StarBase independently. According to the results obtained from GEPIA (Figure 6), RP11-54H7.4, and LINC00152 are related to poor overall survival in patients with HNSC, while according to StarBase (Figure 7), RP11-54H7.4 and LINC01405 are associated with a poor prognosis for patients with HNSC. Clinical prognostic information on LINC01405 was unavailable in GEPIA, and details on CTD-2545M3.8 were not accessible in StarBase.

### Construction and Analysis of the lncRNA-TF-mRNA Network

**Figure 8:**
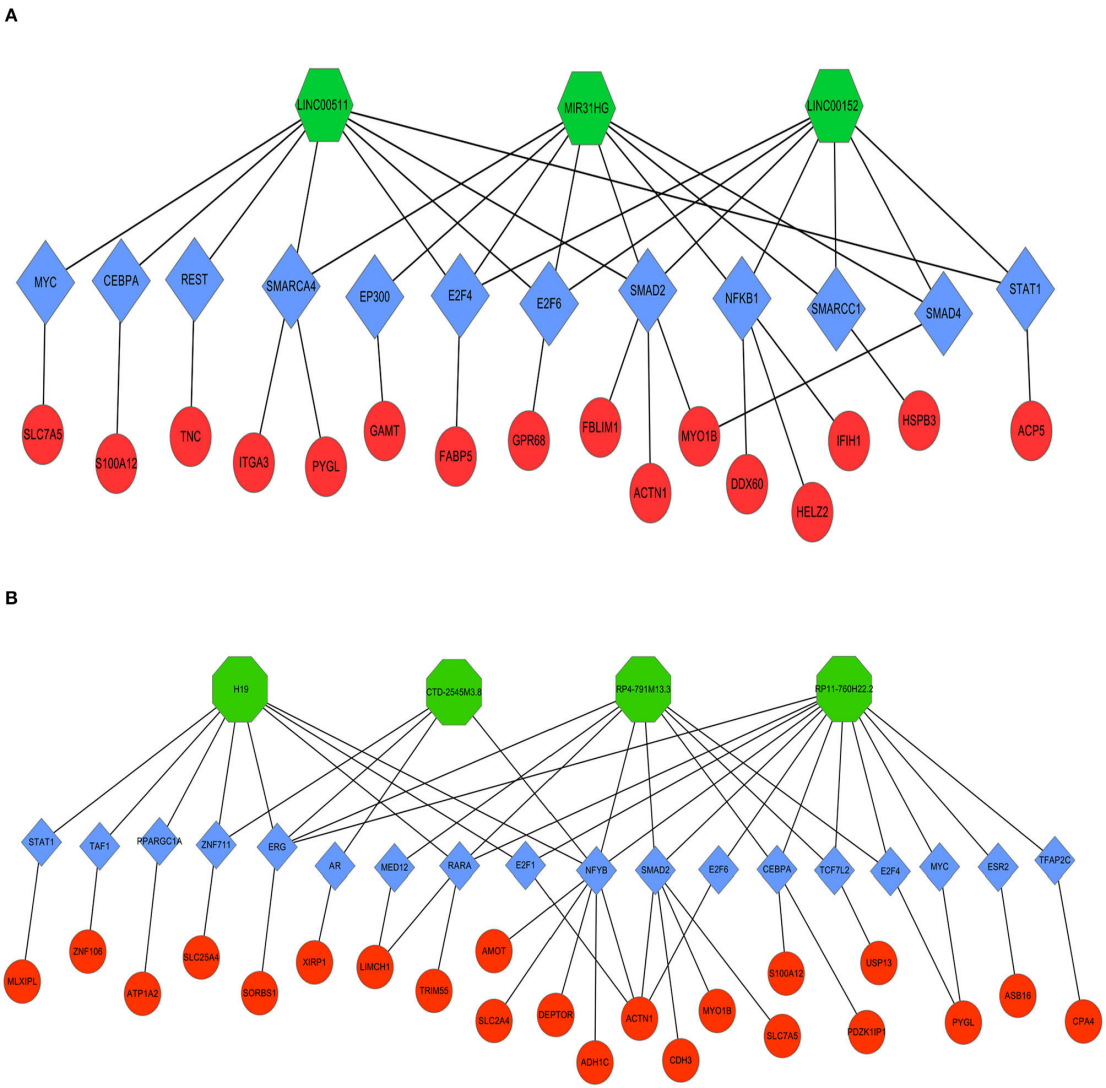
Coexpression regulatory network of lncRNAs-TFs-mRNAs in TSCC. Octagons represent lncRNAs. Diamonds represent TFs. Ellipses represent mRNAs. **(A)** Upregulated DELs and **(B)** Downregulated DELs.

Next, LncMAP was applied to determine lncRNA-TF-mRNA triplets in TSCC. Seven of the nine pivotal DELs associated with TFs were traced. Venn diagrams were used to identify 33 overlapping mRNAs (i.e., overlapping mRNA in triplets and DEGs; Table 3). The expression of the intersecting mRNAs was further confirmed by GEPIA. The expression of S100A12, HSPB3, GAMT, FABP5, ASB16, CPA4, LIMCH1, MLXIPL, PDZK1IP1, TRIM55, USP13, XIRP1, and ZNF106 was not significantly different between HNSC and normal tissues (Supplementary Figures 1, 2). Moreover, the coexpression of TFs and overlapping mRNAs was analyzed with StarBase (Supplementary Figures 3, 4). Pairs of closely related TFs-mRNAs were used to build a regulatory network. Ultimately, we successfully constructed and visualized the lncRNA-TF-mRNA network, including the upregulated (Figure 8A) and downregulated (Figure 8B) DELs, with Cytoscape.

**Table 3:** Intersecting mRNAs between mRNA in triplets and DEGs.

**Ensembl ID**	**LncRNA**	**TF symbol**	**Gene symbol**
	**symbol**		
ENST00000304425	MIR31HG	SMAD2	FBLIM1, ACTN1, MYO1B
		E2F6	GPR68
		E2F4	FABP5
		NFKB1	DDX60, HELZ2, IFIH1
		SMARCC1	HSPB3
		SMAD4	MYO1B
		SMARCA4	ITGA3, PYGL
		EP300	GAMT
ENST00000331944	LINC00152	SMAD2	FBLIM1, ACTN1, MYO1B
		E2F6	GPR68
		E2F4	FABP5
		NFKB1	DDX60, HELZ2, IFIH1
		SMARCC1	HSPB3
		STAT1	ACP5
ENST00000453722	LINC00511	SMAD2	FBLIM1, ACTN1, MYO1B
ENST00000457958			
		E2F6	GPR68
		E2F4	FABP5
		SMARCA4	ITGA3, PYGL
		STAT1	ACP5
		REST	TNC
		CEBPA	S100A12
		MYC	SLC7A5
ENST00000446406	H19	ERG	SORBS1
		NFYB	AMOT, SLC2A4, DEPTOR, ADH1C, ACTN1
		RARA	TRIM55, LIMCH1
		E2F1	ACTN1
		PPARGC1A	ATP1A2
		TAF1	ZNF106
		STAT1	MLXIPL
ENST00000595005	CTD-	ERG	SORBS1
	2545M3.8	NFYB	AMOT, SLC2A4, DEPTOR, ADH1C, ACTN1
		ZNF711	SLC25A4
		AR	XIRP1
ENST00000466343	RP4-	ERG	SORBS1
	791M13.3	NFYB	AMOT, SLC2A4, DEPTOR, ADH1C, ACTN1
		RARA	TRIM55, LIMCH1
		SMAD2	CDH3, MYO1B, SLC7A5, ACTN1
		CEBPA	S100A12, PDZK1IP1
		TCF7L2	USP13
		E2F4	PYGL
		MED12	LIMCH1
ENST00000520544	RP11-	ERG	SORBS1
	760H22.2	NFYB	AMOT, SLC2A4, DEPTOR, ADH1C, ACTN1
		RARA	TRIM55, LIMCH1
		E2F1	ACTN1
		SMAD2	CDH3, MYO1B, SLC7A5, ACTN1
		CEBPA	S100A12, PDZK1IP1
		TCF7L2	USP13
		E2F4	PYGL
		ESR2	ASB16
		MYC	PYGL
		E2F6	ACTN1
		TFAP2C	CPA4

### Screening Biomarkers

**Figure 9:**
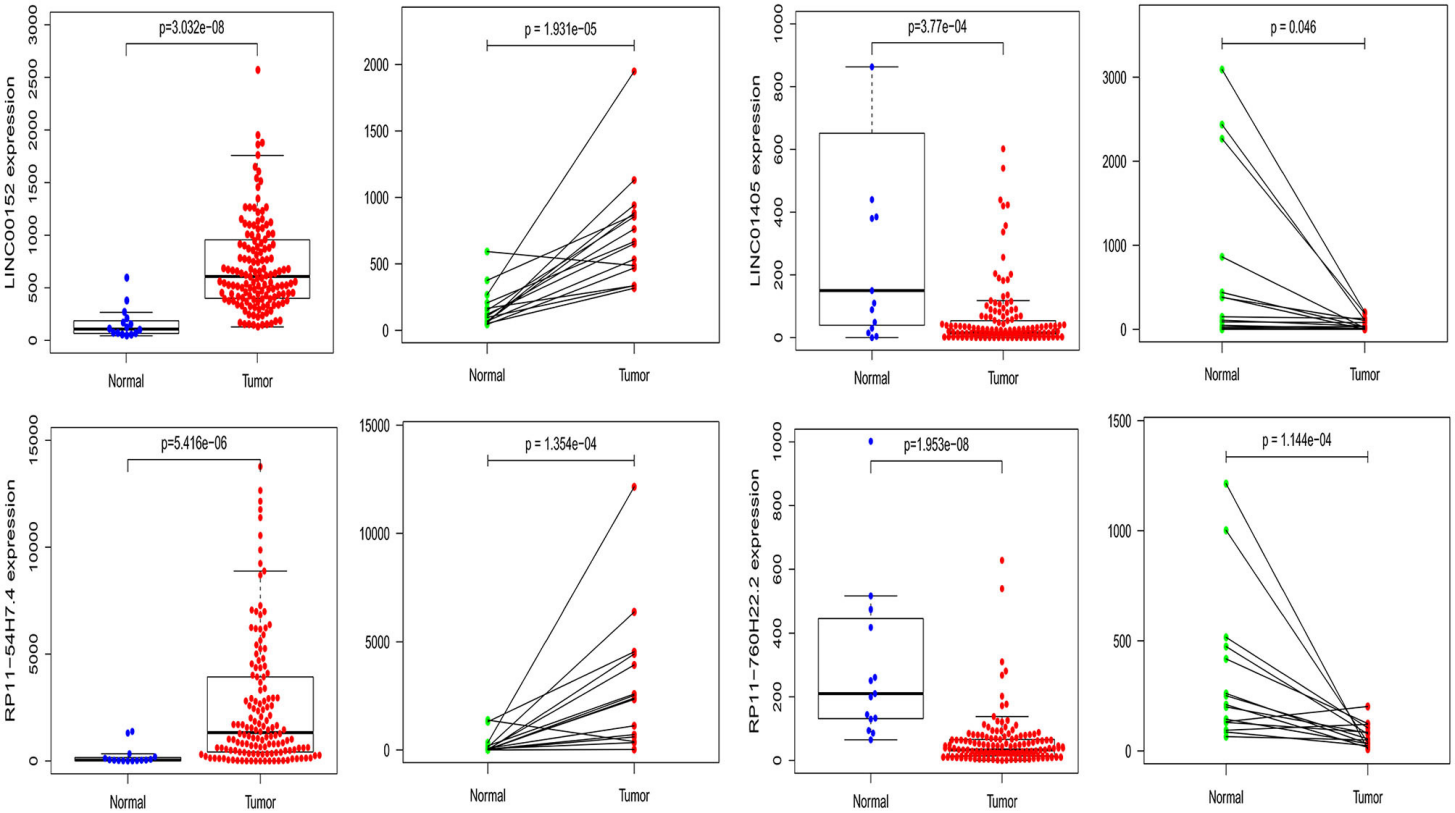
The expression of RP11-54H7.4, LINC00152, LINC01405, and RP11-760H22.2 were then analyzed in the TSCC and normal tissues, and their expression in the paired groups.

**Figure 10:**
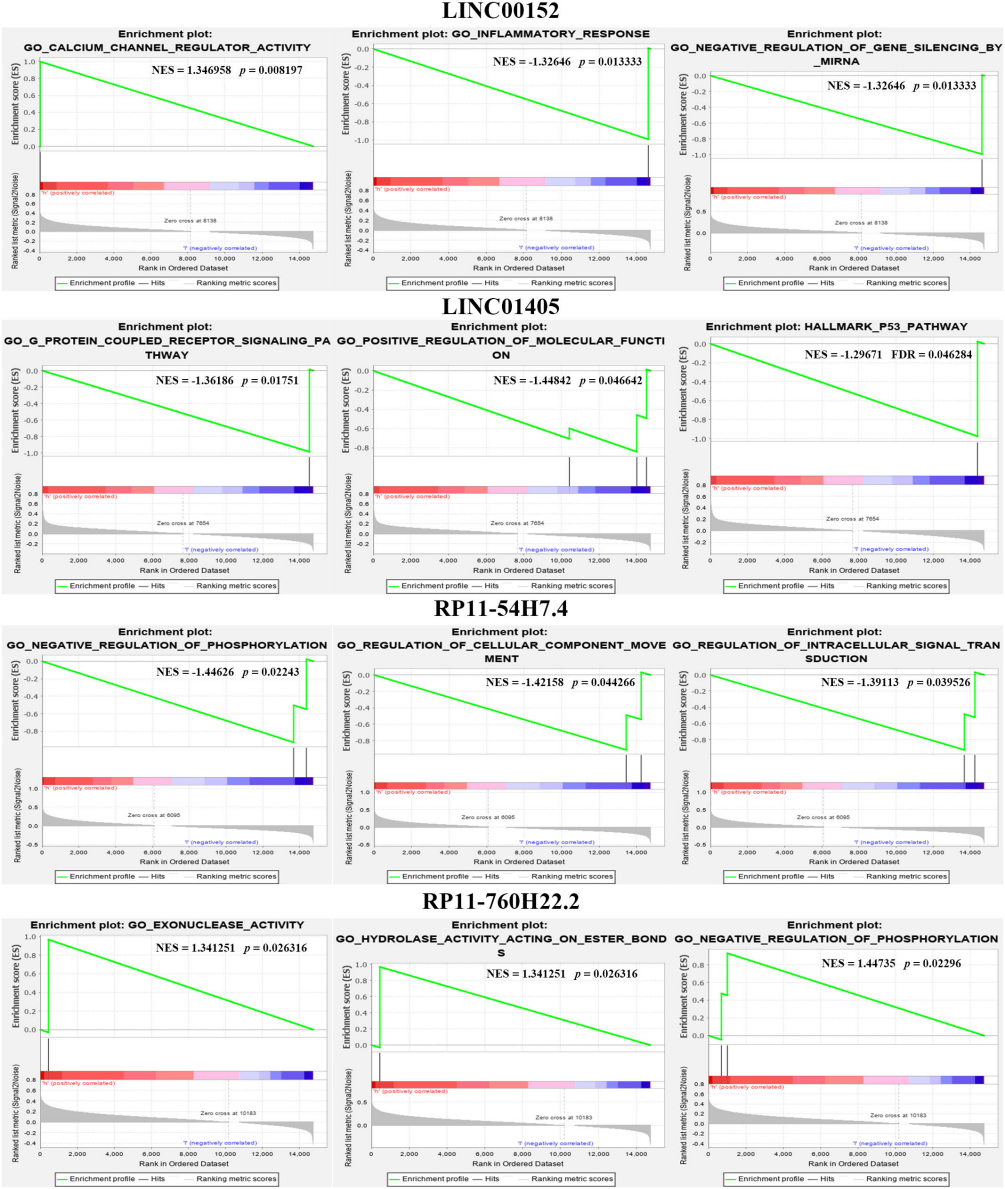
Several pivotal GSEA results of RP11-54H7.4, LINC00152, LINC01405, and RP11-760H22.2 were performed by GSEA software independently.

Furthermore, we also compared our findings with the DELs screened by TCGA database. As expected, several biomarker expression levels in our results were consistent with TCGA. A few DELs worth noting are RP11-54H7.4, LINC00152, LINC01405, and RP11-760H22.2. The expression of RP11-54H7.4, LINC00152, LINC01405, and RP11-760H22.2 were then analyzed in the TSCC and normal tissues, and their expression in the paired groups (Figure 9). Moreover, several pivotal GSEA results of them were performed by GSEA software independently (Figure 10).

### Validation of the Candidate Biomarkers

**Figure 11:**
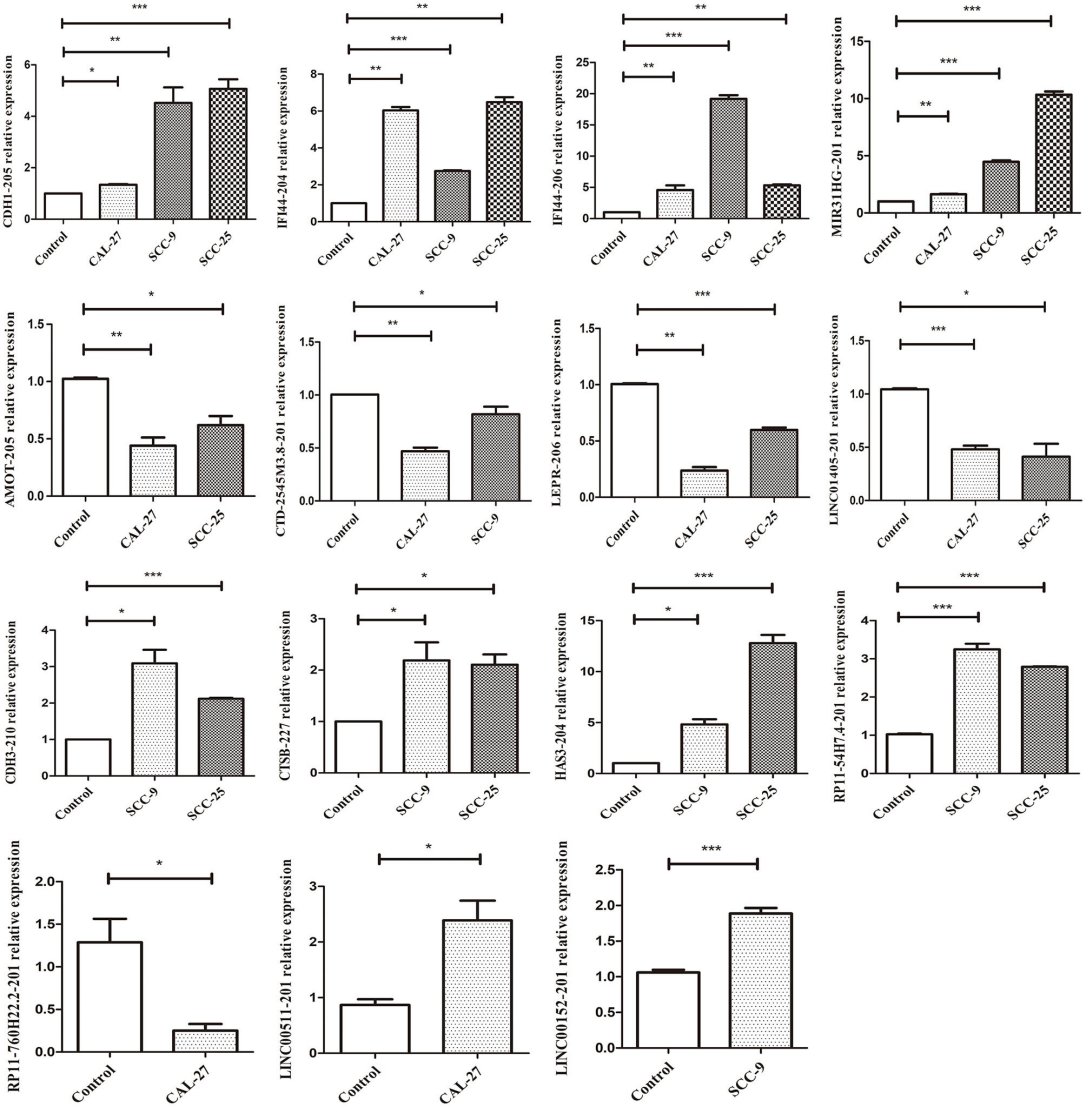
qRT-PCR validation of significant DELs from sequencing (***p < 0.001, **p < 0.01, and *p < 0.05).

qRT-PCR was performed to verify the expression levels of the candidate DELs in TSCC cell lines. The expression of CDH1-205, MIR31HG, IFI44-204, IFI44-206, RP11-54H7.4, CTSB-227, HAS3-204, CDH3-210, LEPR-206, AMOT-205, CTD-2545M3.8, LINC01405, RP11-760H22.2, LINC00152, and LINC00511 was consistent with the sequencing results (Figure 11). All primers information are listed in Supplementary Table 3.

## Discussion

Various studies have demonstrated that lncRNAs are abnormally expressed in diverse tumor types and have broad application prospects in cancer diagnosis, monitoring, prognosis, and treatment response prediction (7, 16, 17).

TSCC is the most common type of OSCC, and its pathogenesis involves the dysregulation of gene networks, including both protein-coding genes and non-coding RNAs (6–8). To date, multiple lncRNAs have been identified in TSCC, providing new perspectives for exploring the molecular mechanism of TSCC pathogenesis (18). In the current study, we sought to screen aberrantly expressed lncRNAs by performing RNA-seq analysis of TSCC tumor tissues. These data indicate that 51,349 transcripts, 38,567 of which are protein coding, 4,224 mRNAs and 1,470 lncRNAs are differentially expressed between TSCC tumor and adjacent tissues. Through data preprocessing and limma package analysis, we identified 40 candidate DELs in TSCC. Then, 15 dysregulated lncRNAs were further verified using qRT-PCR, the results may be related to the heterogeneity between different TSCC cell lines. We also selected nine pivotal lncRNAs through an online public database. Among them, in TSCC, RP11-54H7.4, MIR31HG, LINC00152, and LINC00511 were upregulated, while H19, CTD-2545M3.8, RP11-760H22.2, RP4-791M13.3, and LINC01405 were consistently downregulated. The most well-known lncRNA, MIR31HG, can act as a HIF-1α coactivator to modulate hypoxia signaling pathways in oral cancer (17). It has also been reported that the lncRNA MIR31HG facilitates HNSC cell proliferation and tumorigenesis via HIF1A and p21 by promoting cell cycle progression and inhibiting cell apoptosis (19). A previous study showed that LINC00152 might promote the growth and invasion of OSCC by regulating miR-139-5p (20). The same study proved that LINC00511 could modulate TSCC progression (21). Various findings have revealed that H19 plays a critical role in the regulation of TSCC migration and invasion (22, 23). Our results are consistent with those of previous studies, and we also found that there is limited research on RP11-54H7.4, CTD-2545M3.8, RP11-760H22.2, RP4-791M13.3, and LINC01405 in TSCC pathogenesis.

GO analysis of the DELs revealed that some biological processes and molecular functions, such as cellular respiration, and oxidative phosphorylation could be involved in the progression of TSCC. Annotation of the most significant KEGG pathways associated with the DELs manifested the important pathways that could play pivotal roles in the mechanism of TSCC tumorigenesis, including oxidative phosphorylation, RNA transport, and mRNA surveillance pathway, suggesting that dysregulated lncRNAs may have a great influence on these targets by regulating associated pathways in TSCC. However, the functions of most lncRNAs are not well-understood.

The most significant GO terms of the DEGs were immune system processes, the immune response, and protein binding. KEGG pathway analysis of the DEGs revealed 20 pathways that could play pivotal roles in the mechanism of TSCC tumorigenesis, including the cell cycle, metabolic, and NOD-like receptor signaling pathway. The results suggest that these pathways might contribute significantly to the pathogenesis and progression of TSCC.

This study found that RP11-54H7.4, LINC00152, and LINC01405 are significantly aberrantly expressed based on the RNA-seq expression profile and associated with poor clinical outcomes in TSCC patients, the results are consistent with prior studies (24, 25). The expression of these lncRNAs was further confirmed in GEPIA and StarBase. Besides, the expression of RP11-54H7.4, LINC00152, LINC01405, and RP11-760H22.2 were also verified with TSCC samples in TCGA. Thus, RP11-54H7.4, LINC00152, and LINC01405 could represent prognostic biomarkers of TSCC.

Research shows that lncRNAs might play a role in trans-acting regulation via TFs, while the interactions between lncRNAs and TFs may ameliorate the expression levels of their target genes (26). The expression of target genes could also be regulated by TFs combining cis-elements at promoter locations (27). It is known that some TFs are involved in TSCC pathogenesis. To further investigate the functions of DELs in TSCC, seven of nine pivotal DELs associated TFs, namely, SMAD2, E2F6, E2F4, CEBPA, STAT1, MYC, ERG, and NFYB, were further analyzed. Research has demonstrated that SMAD2 acts as a predictor of overall survival in OSCC patients (28). Additionally, SMAD2 directly binds to the lncRNA MACC1-AS1 promoter and thus increases MACC1-AS1 expression in nasopharyngeal carcinoma (29). Studies have also shown that silencing the lncRNA CEBPA-AS1 can regulate OSCC cell proliferation, cell apoptosis, migration, and invasion by targeting CEBPA and via a novel pathway, CEBPA/Bcl2 (30). Similarly, abundant studies demonstrated that E2F4, STAT1, MYC, ERG, and NFYB may play an important role in the pathogenesis and progression of various cancers, including OSCC, and the underlying mechanism may be related to dysregulated lncRNA (31–37). All of these studies confirm that the TFs screened in our research play a critical role in tumor pathogenesis. However, the underlying mechanisms remain unknown. Therefore, we hypothesized that these TFs play vital roles in the tumorigenesis of TSCC by regulating lncRNAs and mRNAs. In addition, the relationship between lncRNAs and TFs requires further investigation.

The overlapping genes were screened by comparing the mRNA in triplets and DEGs. Some vital mRNAs, including MYO1B, ACTN1, PYGL, S100A12, and SLC7A5, are associated with TSCC (38–40). We also found that many TF expression levels were significantly correlated with the expression of multiple protein-coding genes. We thus deduce that these TFs might be correlated with the carcinogenesis of TSCC by regulating coexpressing genes. Therefore, a coexpression network was built to further investigate the relationship of the dysregulated TFs and mRNAs. We were able to successfully build the lncRNA-TF-mRNA network by combining all the results. The lncRNA-TF-mRNA coexpression network was constructed to predict lncRNA function. More critically, the interaction network of the significantly dysregulated lncRNAs with their TFs and coding genes was delineated, which might provide new clues for elucidating the underlying mechanism of TSCC.

## Conclusion

To conclude, we found a profile of dysregulated lncRNAs, TFs, and mRNAs that could serve as prospective clinical biomarkers because of their tissue specificity and association with the tumorigenesis and progression of TSCC. Our study might lay a foundation for further functional research on lncRNAs-TFs-mRNAs in TSCC. The composite analysis of lncRNAs, TFs, and differentially coexpressed genes may provide a more comprehensive understanding of the pathogenesis of TSCC. Our results also suggest that specific lncRNAs, TFs, and mRNAs could be valuable for the diagnosis and treatment of tongue cancer, contribute to the application of lncRNAs in cancer and provide deep insights into the biological mechanism of TSCC. Intriguingly, this is the first study to show that RP11-54H7.4, LINC00152, and LINC01405 can be acted as a prognostic target for TSCC.

Similar to other approaches, our study also had certain limitations. First, in the survival analysis, because of the lack of clinical data that matched our data, it was difficult to further validate some prognosis-related lncRNAs. Furthermore, the interaction between lncRNAs, TFs, and mRNAs still lacks experimental verification. These deficiencies will be improved by complementing these data in the future.

## Data Availability Statement

The datasets presented in this study can be found in online repositories. The names of the repository/repositories and accession number(s) can be found below: https://www.ncbi.nlm.nih.gov/geo/, GSE149008/b.

## Ethics Statement

The studies involving human participants were reviewed and approved by Ethics Committee of the First Affiliated Hospital of Fujian Medical University. The patients/participants provided their written informed consent to participate in this study.

## Author Contributions

MZ: research design and implementation. ZC, YL, and YH: data analysis and interpretation. SZ, YJ, and LW: statistical analysis. MZ: writing of the manuscript. JC and BS: revision of the manuscript. All authors contributed to the article and approved the submitted version.

## Conflict of Interest

The authors declare that the research was conducted in the absence of any commercial or financial relationships that could be construed as a potential conflict of interest.

## Abbreviations

DEL: differentially expressed lncRNA
DEG: differentially expressed mRNA
LncRNA: long non-coding RNA
TF: transcription factor
TSCC: tongue squamous cells carcinomas
HNSC: head and neck squamous cell carcinoma
GO: Gene Ontology
KEGG: Kyoto Encyclopedia of Genes and Genomes.
