# Supplementary material for: Analysis of Differentially Expressed Long Non-coding RNAs and the Associated TF-mRNA Network in Tongue Squamous Cell Carcinoma
Source: Front Oncol. 2020 Aug 14;10:1421. doi: 10.3389/fonc.2020.01421 (PMC7456846; doi:10.3389/fonc.2020.01421)
Supplement: Supplementary file 1 [file Data_Sheet_1.docx]

**Supplementary Material**

**Supplementary Table 1** Clinical characteristics of the six enrolled patients

| number | Sex | Age at surgery(years) | Tumor size(cm) | TNM | Histological grade |
| --- | --- | --- | --- | --- | --- |
| C1 | Female | 51 | 3.2 | T3N0M0 | High |
| C2 | Male | 65 | 1.5 | T2N0M0 | High |
| C3 | Male | 45 | 3.5 | T2N0M0 | High |
| C4 | Male | 53 | 2.5 | T4aN2M0 | Middle |
| C5 | Male | 43 | 2.0 | T2N2M0 | High |
| C6 | Male | 48 | 3.0 | T2N0M0 | Middle |

**Supplementary Table 2 The GSEA results of four-lncRNA**

| **lncRNA** | **GS follow link to MSIGDB** | **SIZE** | **ES** | **NES** | **NOM p-val** | **FDR q-val** |
| --- | --- | --- | --- | --- | --- | --- |
| LINC00152 | GO_CHANNEL_REGULATOR_ACTIVITY | 1 | 0.997491 | 1.346958 | 0.008197 | 1 |
|  | GO_CALCIUM_CHANNEL_REGULATOR_ACTIVITY | 1 | 0.997491 | 1.346958 | 0.008197 | 1 |
|  | GO_ION_CHANNEL_REGULATOR_ACTIVITY | 1 | 0.997491 | 1.346958 | 0.008197 | 1 |
|  | GO_SINGLE_FERTILIZATION | 1 | 0.978635 | 1.302637 | 0.023158 | 1 |
|  | GO_MEMBRANE_FUSION | 1 | 0.978635 | 1.302637 | 0.023158 | 1 |
|  | GO_SEXUAL_REPRODUCTION | 1 | 0.978635 | 1.302637 | 0.023158 | 1 |
|  | GO_CELLULAR_PROCESS_INVOLVED_IN_REPRODUCTION_IN_MULTICELLULAR_ORGANISM | 1 | 0.978635 | 1.302637 | 0.023158 | 1 |
|  | GO_MULTI_ORGANISM_REPRODUCTIVE_PROCESS | 1 | 0.978635 | 1.302637 | 0.023158 | 1 |
|  | GO_PLASMA_MEMBRANE_FUSION | 1 | 0.978635 | 1.302637 | 0.023158 | 1 |
|  | GO_MEMBRANE_ORGANIZATION | 1 | 0.978635 | 1.302637 | 0.023158 | 1 |
|  | GO_FERTILIZATION | 1 | 0.978635 | 1.302637 | 0.023158 | 1 |
|  | GO_REPRODUCTION | 1 | 0.978635 | 1.302637 | 0.023158 | 1 |
|  | GO_MULTICELLULAR_ORGANISM_REPRODUCTION | 1 | 0.978635 | 1.302637 | 0.023158 | 1 |
|  | GO_FUSION_OF_SPERM_TO_EGG_PLASMA_MEMBRANE_INVOLVED_IN_SINGLE_FERTILIZATION | 1 | 0.978635 | 1.302637 | 0.023158 | 1 |
|  | GO_CELL_MOTILITY | 2 | 0.90619 | 1.464428 | 0.037328 | 1 |
|  | GO_LOCOMOTION | 2 | 0.90619 | 1.464428 | 0.037328 | 0.6605 |
|  | GO_REGULATION_OF_CELLULAR_COMPONENT_MOVEMENT | 2 | 0.90619 | 1.464428 | 0.037328 | 0.440334 |
|  | GO_REGULATION_OF_GTPASE_ACTIVITY | 2 | -0.95186 | -1.51114 | 0.009542 | 0.105536 |
|  | GO_ENZYME_ACTIVATOR_ACTIVITY | 2 | -0.95186 | -1.51114 | 0.009542 | 0.079152 |
|  | GO_NUCLEOSIDE_TRIPHOSPHATASE_REGULATOR_ACTIVITY | 2 | -0.95186 | -1.51114 | 0.009542 | 0.063321 |
|  | GO_POSITIVE_REGULATION_OF_GTPASE_ACTIVITY | 2 | -0.95186 | -1.51114 | 0.009542 | 0.052768 |
|  | GO_GTPASE_REGULATOR_ACTIVITY | 2 | -0.95186 | -1.51114 | 0.009542 | 0.04523 |
|  | GO_INFLAMMATORY_RESPONSE | 1 | -0.99234 | -1.32646 | 0.013333 | 0.389442 |
|  | GO_REGULATION_OF_INFLAMMATORY_RESPONSE | 1 | -0.99234 | -1.32646 | 0.013333 | 0.361625 |
|  | GO_POSITIVE_REGULATION_OF_INFLAMMATORY_RESPONSE | 1 | -0.99234 | -1.32646 | 0.013333 | 0.337516 |
|  | GO_MIRNA_BINDING | 1 | -0.99234 | -1.32646 | 0.013333 | 0.316422 |
|  | GO_RIBONUCLEOPROTEIN_COMPLEX_BINDING | 1 | -0.99234 | -1.32646 | 0.013333 | 0.297808 |
|  | GO_REGULATORY_RNA_BINDING | 1 | -0.99234 | -1.32646 | 0.013333 | 0.281264 |
|  | GO_NEGATIVE_REGULATION_OF_GENE_SILENCING_BY_MIRNA | 1 | -0.99234 | -1.32646 | 0.013333 | 0.26646 |
|  | GO_POSITIVE_REGULATION_OF_CELL_POPULATION_PROLIFERATION | 4 | -0.8645 | -1.5792 | 0.017613 | 0.086961 |
|  | GO_REGULATION_OF_CELL_POPULATION_PROLIFERATION | 4 | -0.8645 | -1.5792 | 0.017613 | 0.043481 |
| LINC01405 | GO_NUCLEAR_OUTER_MEMBRANE_ENDOPLASMIC_RETICULUM_MEMBRANE_NETWORK | 2 | -0.9922 | -1.33064 | 0.011321 | 0.577555 |
|  | GO_OXIDATION_REDUCTION_PROCESS | 1 | -0.9922 | -1.33063 | 0.011321 | 0.551302 |
|  | GO_VESICLE_MEMBRANE | 1 | -0.9922 | -1.33063 | 0.011321 | 0.527333 |
|  | GO_OXIDOREDUCTASE_ACTIVITY_OXIDIZING_METAL_IONS | 1 | -0.9922 | -1.33063 | 0.011321 | 0.50536 |
|  | GO_COFACTOR_BINDING | 1 | -0.9922 | -1.33063 | 0.011321 | 0.485146 |
|  | GO_TETRAPYRROLE_BINDING | 1 | -0.9922 | -1.33063 | 0.011321 | 0.466486 |
|  | GO_OXIDOREDUCTASE_ACTIVITY | 1 | -0.9922 | -1.33063 | 0.011321 | 0.449209 |
|  | GO_OXIDOREDUCTASE_ACTIVITY_OXIDIZING_METAL_IONS_OXYGEN_AS_ACCEPTOR | 1 | -0.9922 | -1.33063 | 0.011321 | 0.433166 |
|  | GO_DEFENSE_RESPONSE | 4 | -0.80819 | -1.58292 | 0.012097 | 0.230463 |
|  | GO_G_PROTEIN_COUPLED_RECEPTOR_SIGNALING_PATHWAY | 1 | -0.98596 | -1.36186 | 0.01751 | 0.850852 |
|  | GO_GTPASE_ACTIVITY | 1 | -0.98596 | -1.36186 | 0.01751 | 0.756313 |
|  | GO_HYDROLASE_ACTIVITY_ACTING_ON_ACID_ANHYDRIDES | 1 | -0.98596 | -1.36186 | 0.01751 | 0.680682 |
|  | GO_REGULATION_OF_G_PROTEIN_COUPLED_RECEPTOR_SIGNALING_PATHWAY | 1 | -0.98596 | -1.36186 | 0.01751 | 0.618802 |
|  | GO_RESPONSE_TO_BIOTIC_STIMULUS | 3 | -0.82203 | -1.4544 | 0.034682 | 0.64275 |
|  | GO_ENDOPLASMIC_RETICULUM | 2 | -0.88578 | -1.36078 | 0.03992 | 0.576535 |
|  | GO_POSITIVE_REGULATION_OF_HYDROLASE_ACTIVITY | 3 | -0.84188 | -1.44842 | 0.046642 | 0.511583 |
|  | GO_POSITIVE_REGULATION_OF_CATALYTIC_ACTIVITY | 3 | -0.84188 | -1.44842 | 0.046642 | 0.409266 |
|  | GO_POSITIVE_REGULATION_OF_MOLECULAR_FUNCTION | 3 | -0.84188 | -1.44842 | 0.046642 | 0.341055 |
|  | HALLMARK_P53_PATHWAY | 1 | -0.97592 | -1.29671 | 0.062619 | 0.046284 |
| RP11-54H7.4 | GO_NEGATIVE_REGULATION_OF_PHOSPHORYLATION | 2 | -0.9279 | -1.44626 | 0.02243 | 1 |
|  | GO_REGULATION_OF_PHOSPHORYLATION | 2 | -0.9279 | -1.44626 | 0.02243 | 0.52685 |
|  | GO_REGULATION_OF_INTRACELLULAR_SIGNAL_TRANSDUCTION | 2 | -0.9279 | -1.39113 | 0.039526 | 0.321743 |
|  | GO_CELL_MOTILITY | 2 | -0.91243 | -1.42158 | 0.044266 | 0.482154 |
|  | GO_LOCOMOTION | 2 | -0.91243 | -1.42158 | 0.044266 | 0.361616 |
|  | GO_REGULATION_OF_CELLULAR_COMPONENT_MOVEMENT | 2 | -0.91243 | -1.42158 | 0.044266 | 0.289293 |
| RP11-760H22.2 | GO_NEGATIVE_REGULATION_OF_PHOSPHORUS_METABOLIC_PROCESS | 3 | 0.878917 | 1.55002 | 0.008333 | 0.360647 |
|  | GO_REGULATION_OF_PHOSPHORUS_METABOLIC_PROCESS | 3 | 0.878917 | 1.55002 | 0.008333 | 0.180324 |
|  | GO_NEGATIVE_REGULATION_OF_PHOSPHORYLATION | 2 | 0.931289 | 1.447353 | 0.022965 | 0.607042 |
|  | GO_REGULATION_OF_PHOSPHORYLATION | 2 | 0.931289 | 1.447353 | 0.022965 | 0.455281 |
|  | GO_NUCLEIC_ACID_PHOSPHODIESTER_BOND_HYDROLYSIS | 1 | 0.969547 | 1.341251 | 0.026316 | 0.821226 |
|  | GO_HYDROLASE_ACTIVITY_ACTING_ON_ESTER_BONDS | 1 | 0.969547 | 1.341251 | 0.026316 | 0.746569 |
|  | GO_NUCLEASE_ACTIVITY | 1 | 0.969547 | 1.341251 | 0.026316 | 0.684355 |
|  | GO_EXONUCLEASE_ACTIVITY | 1 | 0.969547 | 1.341251 | 0.026316 | 0.631713 |

**Supplementary Table 3** All the sequences of lncRNA primers used for qRT-PCR

| Target ID | Primer | sequence (5’ to 3’) |
| --- | --- | --- |
| ENST00000562836-CDH1-205 | Forward  Reverse | CCTTATGATTCTCTGCTCGTGTT  TCAAGTAGTCATAGTCCTGGTCTT |
| ENST00000304425-MIR31HG- | Forward  Reverse | CCAGATCCTAGCCTCCAGTT  AGGGAAGCACCAGAGAAGTT |
| ENST00000467790-IFI44-204 | Forward  Reverse | TGTCTGCCTTGAGAACTTATGAAC  CACCAAAGCCTGATGCGTTA |
| ENST00000472152-IFI44-206 | Forward  Reverse | GGTTCACGTAAATTTCCTCACATC  AATAAAACACATCCCTTTGGTCTT |
| ENST00000618966-AL161431 | Forward  Reverse | TGGGTTCGTTGTGCATGAGA  GGAGGTACGTGTATGACAGCTT |
| ENST00000533110-CTSB-227 | Forward  Reverse | AGACCGTACTCCATCCCTCC  CTGTTTGTAGGTCGGGCTGT |
| ENST00000568321-HAS3-204 | Forward  Reverse | CACAAGTTTCCATGCCGCTG  GACGCACGCTTCTCCATCAA |
| ENST00000569117-CDH3-210 | Forward  Reverse | GCAACTTATGGCTGTAGTGAATG  ACCTGGTGGATGTCTCTTGT |
| ENST00000462765-LEPR-206 | Forward  Reverse | CTGTGCCAACAGCCAAACTC  ATGGTACCAATGGTGGGCTG |
| ENST00000462114-AMOT-205 | Forward  Reverse | AGAGCGTCTAGAGACTGCCA  ATTGGTAGAACGGGCAGTGG |
| ENST00000595005-AC020909 | Forward  Reverse | GTGCGTGGAATCCTCTATTACC  AGGCATTCTGGTCACTGTTC |
| ENST00000331096-LINC01405 | Forward  Reverse | TGAAATGTATCTGGCCCAATCG  AAAGATGTTCCTCGCCCTCT |
| ENST00000520544-AC091563 | Forward  Reverse | CCGTTAGAGCACTGATGACATT  ACACTACTCTGTGGCAGGAAT |
| ENST00000453722-LINC00511-201 | Forward  Reverse | TGATGTCCCAGCACGAGTAG  CCACCCAATTCCCCACTTTC |
| ENST00000331944-LINC00152-201 | Forward  Reverse | TCCAGCACCTCTACCTGTTG  GGACAAGGGATTAAGACACATAGA |
| GAPDH | Forward  Reverse | CACCCACTCCTCCACCTTTGA  TCTCTCTTCCTCTTGTGCTCTTGC |

**Supplementary FIGURE 1** Confirmation of overlapping mRNAs in HNSC by GEPIA. HNSC, head and neck squamous cell carcinoma. (*p*<0.05)

**Supplementary FIGURE 2** (**Continued**) Confirmation of overlapping mRNAs in HNSC by GEPIA. HNSC, head and neck squamous cell carcinoma.

**Supplementary FIGURE 3** Coexpression analysis of TFs and mRNAs in HNSCs analyzed by StarBase. HNSC, head and neck squamous cell carcinoma.

**Supplementary FIGURE 4** (**Continued**) Coexpression analysis of TFs and mRNAs in HNSCs analyzed by StarBase. HNSC, head and neck squamous cell carcinoma.
