# Supplementary figures and images for: Analysis of Differentially Expressed Long Non-coding RNAs and the Associated TF-mRNA Network in Tongue Squamous Cell Carcinoma
Source: Front Oncol. 2020 Aug 14;10:1421. doi: 10.3389/fonc.2020.01421 (PMC7456846; doi:10.3389/fonc.2020.01421)

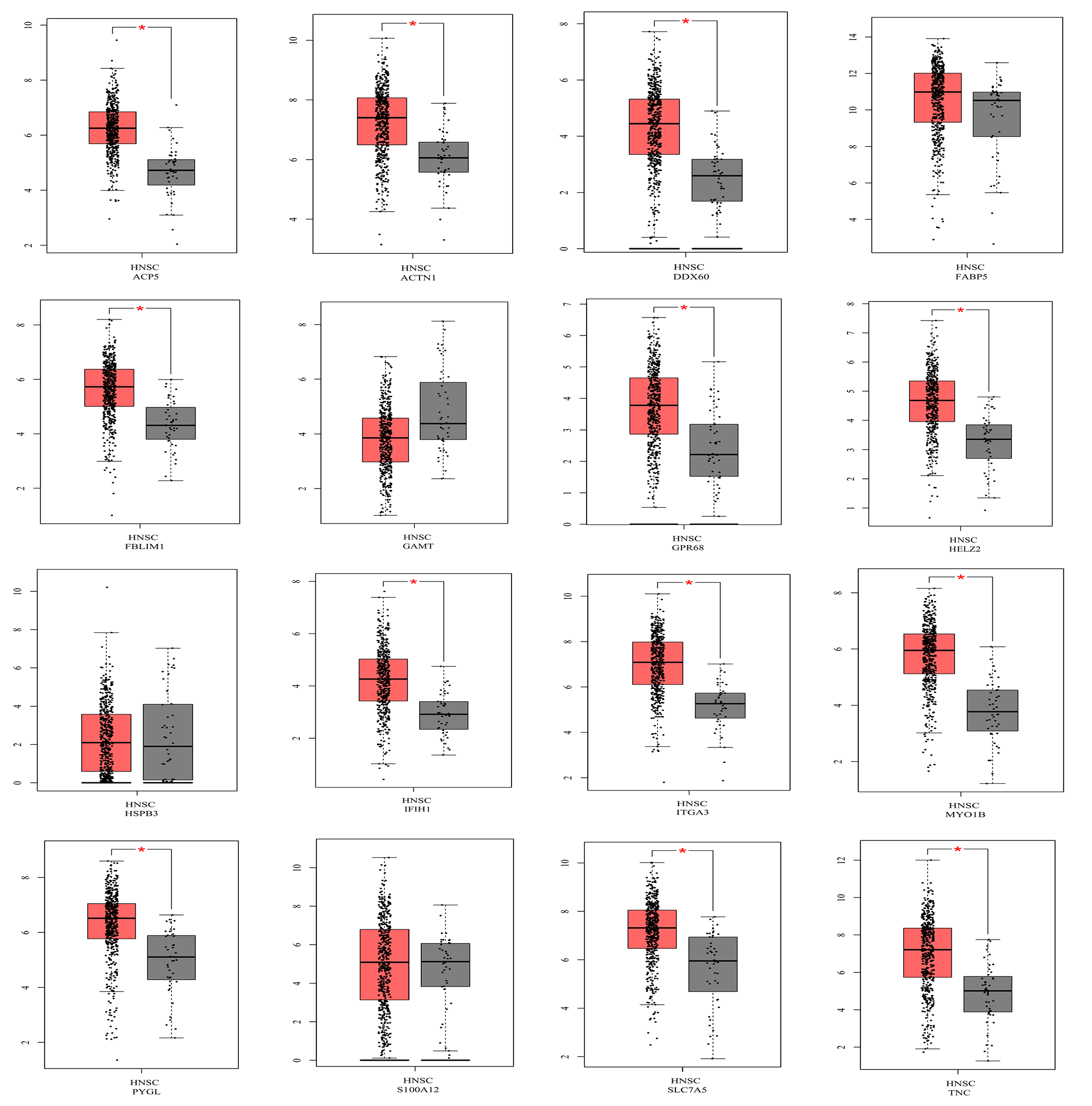

Supplement: Supplementary file 2 [file Image_1.TIF]

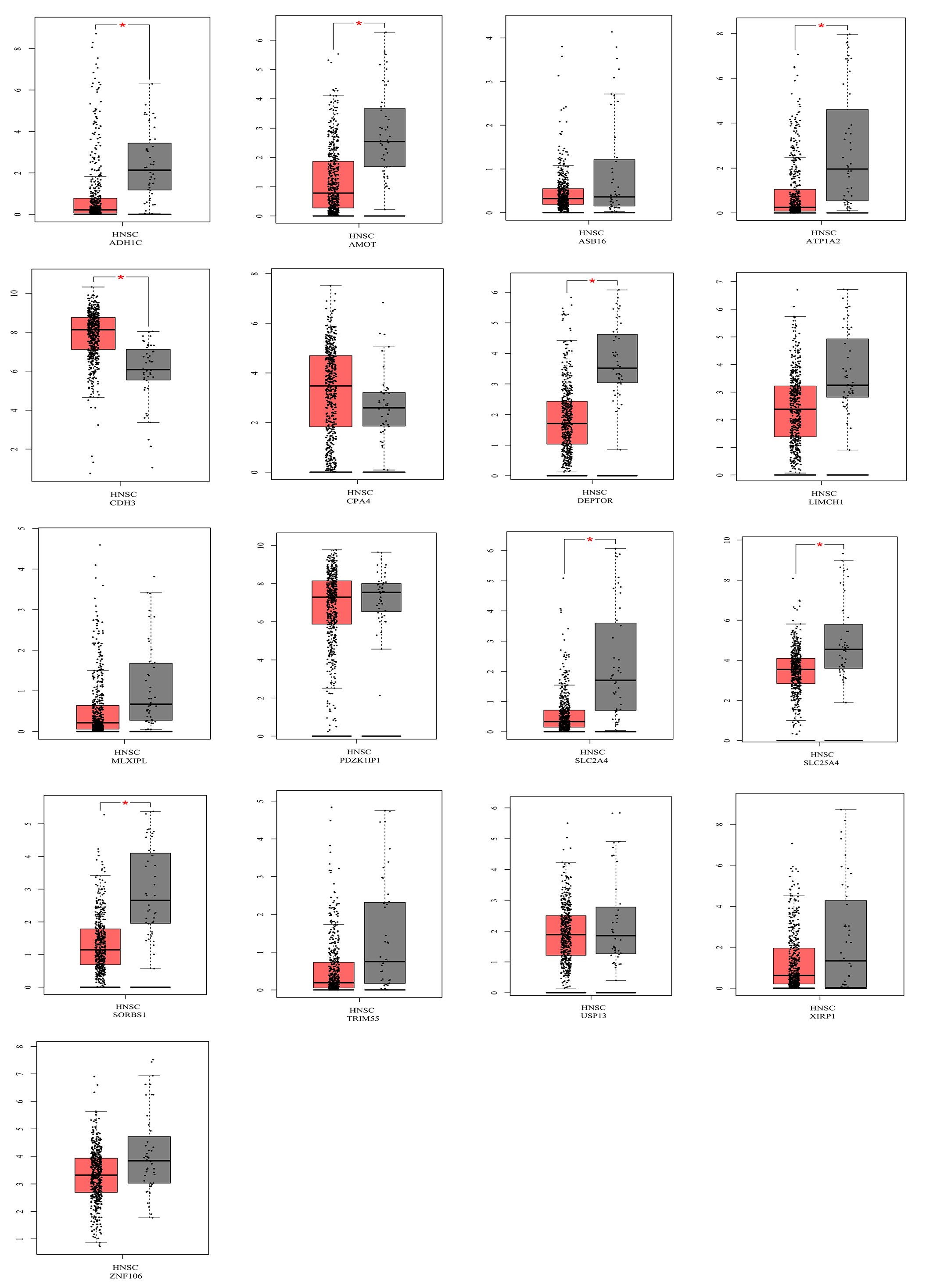

Supplement: Supplementary file 3 [file Image_2.TIF]

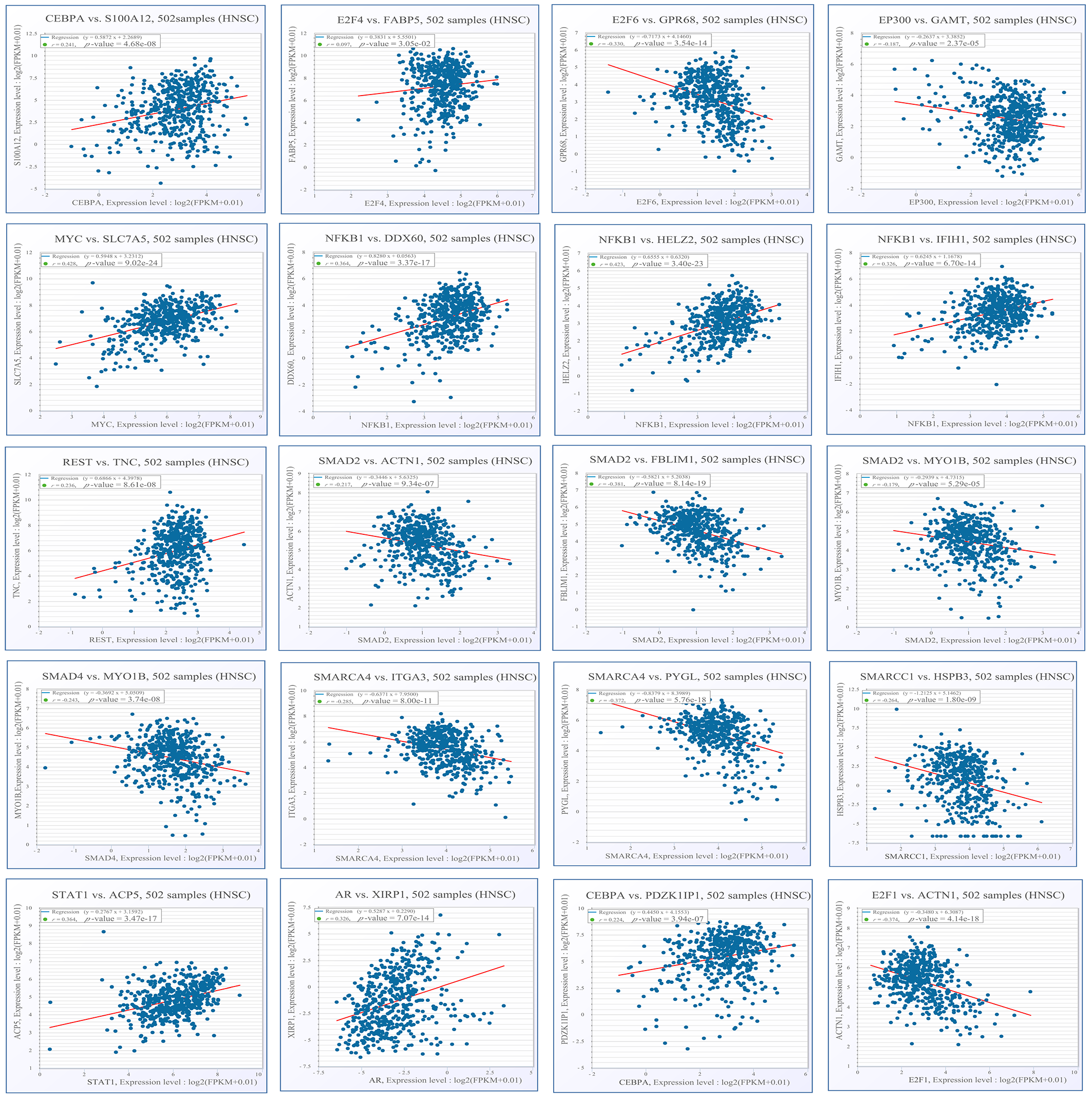

Supplement: Supplementary file 4 [file Image_3.TIF]

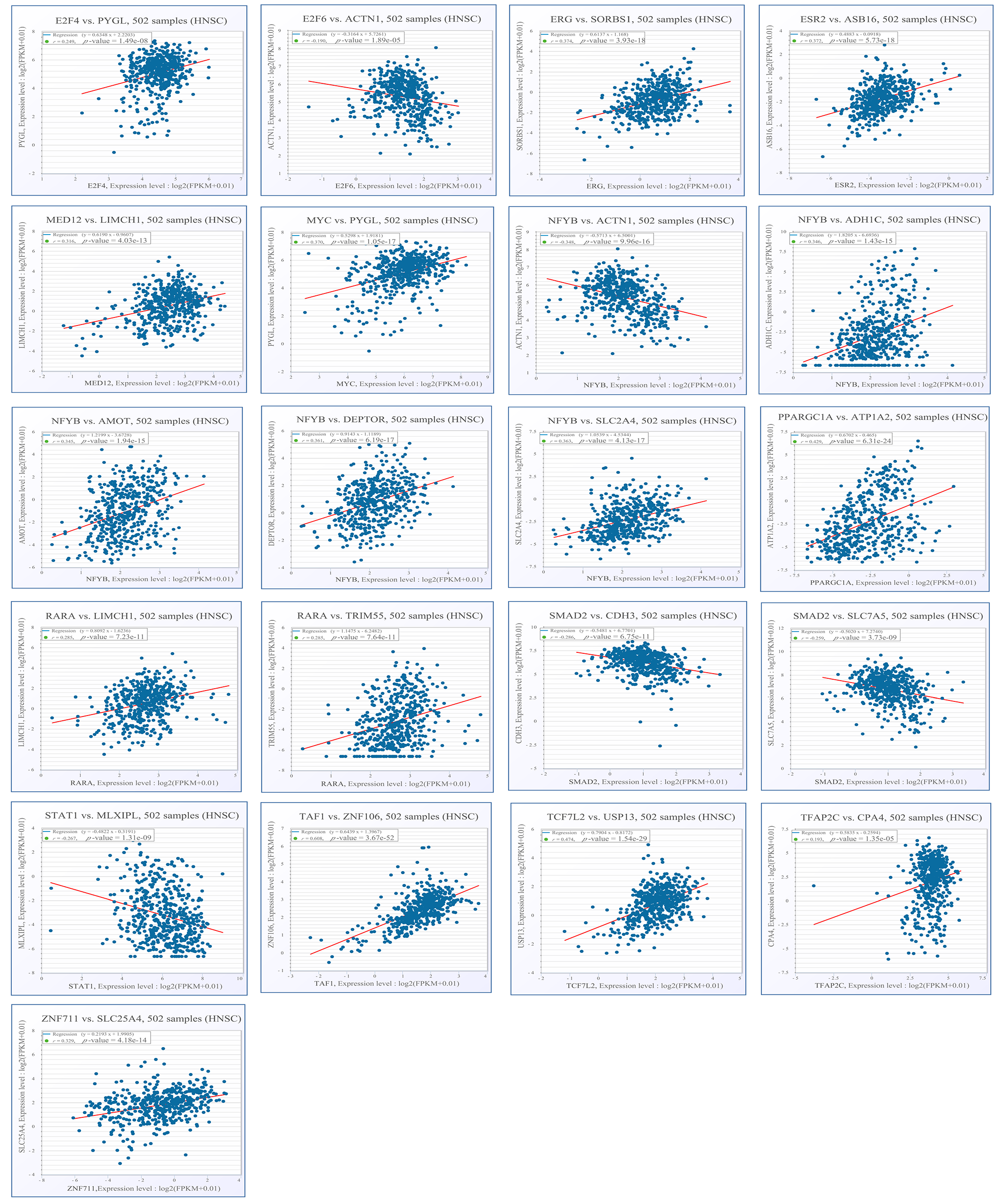

Supplement: Supplementary file 5 [file Image_4.TIF]
